# MicroRNA-encoding long non-coding RNAs

**DOI:** 10.1186/1471-2164-9-236

**Published:** 2008-05-21

**Authors:** Shunmin He, Hua Su, Changning Liu, Geir Skogerbø, Housheng He, Dandan He, Xiaopeng Zhu, Tao Liu, Yi Zhao, Runsheng Chen

**Affiliations:** 1 Bioinformatics Laboratory and National Laboratory of Biomacromolecules, Institute of Biophysics, Chinese Academy of Sciences, Beijing, PR China; 2 Bioinformatics Research Group, Institute of Computing Technology, Chinese Academy of Sciences, Beijing, PR China; 3 Graduate School of the Chinese Academy of Sciences, Beijing, PR China

## Abstract

**Background:**

Recent analysis of the mouse transcriptional data has revealed the existence of ~34,000 messenger-like non-coding RNAs (ml-ncRNAs). Whereas the functional properties of these ml-ncRNAs are beginning to be unravelled, no functional information is available for the large majority of these transcripts.

**Results:**

A few ml-ncRNA have been shown to have genomic loci that overlap with microRNA loci, leading us to suspect that a fraction of ml-ncRNA may encode microRNAs. We therefore developed an algorithm (PriMir) for specifically detecting potential microRNA-encoding transcripts in the entire set of 34,030 mouse full-length ml-ncRNAs. In combination with mouse-rat sequence conservation, this algorithm detected 97 (80 of them were novel) strong miRNA-encoding candidates, and for 52 of these we obtained experimental evidence for the existence of their corresponding mature microRNA by microarray and stem-loop RT-PCR. Sequence analysis of the microRNA-encoding RNAs revealed an internal motif, whose presence correlates strongly (*R*^2 ^= 0.9, *P*-value = 2.2 × 10^-16^) with the occurrence of stem-loops with characteristics of known pre-miRNAs, indicating the presence of a larger number microRNA-encoding RNAs (from 300 up to 800) in the ml-ncRNAs population.

**Conclusion:**

Our work highlights a unique group of ml-ncRNAs and offers clues to their functions.

## Background

The transcriptional output from the genomes of prokaryotic or eukaryotic organisms can be divided into protein-coding mRNAs and non-protein coding RNAs (ncRNAs). Most known ncRNAs are relatively short, but longer messenger-like ncRNAs (ml-ncRNAs) are being detected in increasing numbers [1,2]. Like mRNAs, these RNAs are the products of RNA polymerase II, and are often spliced, capped and polyadenylated [3]. As of now, about one-third of the full-length cDNAs obtained in mice and humans, respectively, appear to be ml-ncRNAs [1,2,4], and several of these have been found to play essential roles *in vivo*. For example, female mice heterozygous for an internal deletion in the Xist gene undergo primary nonrandom inactivation of the wild-type X chromosome, indicating a critical role of Xist RNA for chromosome selection in X inactivation [5]. RNA interference knockdown of the 6.7 kb ncRNA TUG1 in the retina of newborn mice resulted in malformed or nonexistent outer segments of transfected photoreceptors [6], and the activity of the transcription factor NFAT is repressed by the ml-ncRNA repressor NRON [7]. However, most ml-ncRNAs have not yet been characterized, and further elucidation of ml-ncRNA function is an important project for future research on the transcriptome.

MicroRNAs (miRNAs) are usually processed from primary transcripts (pri-miRNAs) to precursor miRNAs (pre-miRNAs) in the nucleus by the RNase III Drosha [8]. Pre-miRNAs are about 70 nt in length and have a stem-loop structure with a 2-nt 3'-overhang [8,9]. The pre-miRNAs are subsequently transported to the cytoplasm by Exportin-5/Ran-GTP, and are further processed by Dicer to produce a ~22 bp duplex miRNA [8,10-14]. The duplex is unraveled by an unidentified RNA helicase and one strand (the mature miRNA) is incorporated into the RNA induced silencing complex (RISC) to guide post-transcriptional gene silencing [15].

Although about the properties of miRNAs are rapidly being unravelled, less is known about the pri-miRNAs. Some pri-miRNAs are thought to be produced by RNA polymerase II, and are capped, polyadenylated and spliced [3,10,16]. The genomic loci of a few ml-ncRNAs overlap with known miRNAs [17], and whole-genome tiling array scans suggest that small RNA loci commonly overlap with longer transcripts, the longer RNAs possibly representing primary transcript of the shorter mature RNAs [18]. The possibility thus exists that a fraction of the existing ml-ncRNAs function as precursors for miRNAs. In this study of mouse ml-ncRNAs, we identified 22 ml-ncRNAs encoding known miRNAs (henceforth labelled miRNA-encoding ncRNAs or me-ncRNAs), and developed a prediction procedure, PriMir, which predicted 97 me-ncRNA candidates among the 34,030 ml-ncRNAs in the FANTOM3 data. For about half of these candidates we obtained experimental evidence for the existence of their corresponding mature miRNA, and further analyses of both known and the candidate me-ncRNAs show that such transcripts frequently share a common motif. Our work specifies me-ncRNAs as a special class of ncRNAs, and suggests a role for these ml-ncRNAs whose functions were previously unidentified.

## Results

### 22 ml-ncRNAs encode known miRNAs

In the mouse genome there are 270 different pre-miRNA hairpins encoding 301 miRNAs (miRBase 8.0 [19]). In order to estimate how many of the 34,030 mouse ml-ncRNAs (FANTOM3 [1]) might encode a known miRNA, we identified the positions of all these ml-ncRNAs and pre-miRNAs in the mouse genome (mm7) using BLAT and Blastn, respectively. The result showed that 23 miRNA hairpins are located in exons of 22 ml-ncRNAs. Of these 22 ml-ncRNAs, three represent overlapping transcripts of different lengths that include the same pre-miRNA stem-loop structure, thus encoding the same miRNA [mmu-mir-22; see Table S1 in Additional file 1].

### Computational analysis identifies strong me-ncRNA candidates

**Figure 1:**
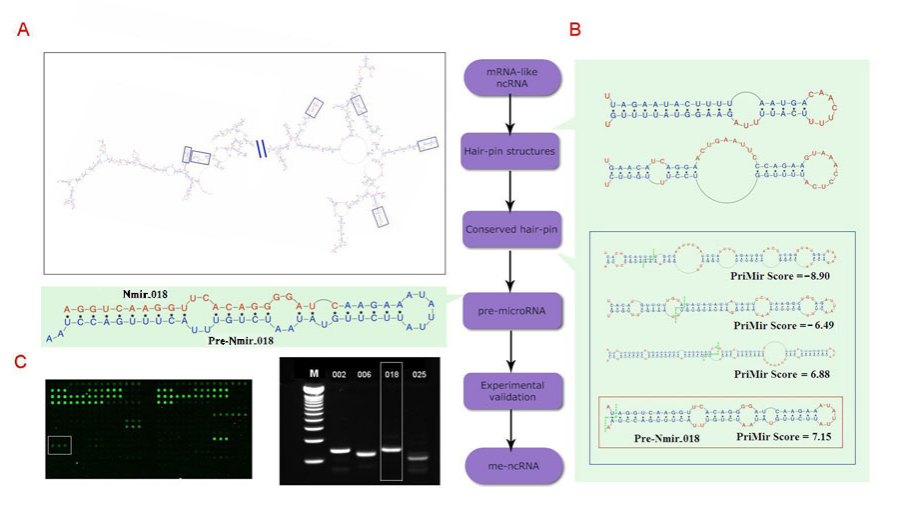
**The PriMir pipeline. Prediction and verification of Nmir_018**. A. The secondary structure of ml-ncRNA A530020N14 as predicted by RNAfold [44, 45]. B. Among the six hairpins extracted from the ml-ncRNA, four are conserved between mouse and rat (blue frame). The green dashed line indicates the pre-miRNA 5' and 3' end positions predicted by PriMir. Of the four conserved hairpins, one had a PriMir score above 7, and was regarded as a pre-miRNA candidate (pre-Nmir_018; red frame). C. The upper part of the panel shows the predicted pre-Nmir_018, red color letters indicating the position of the mature miRNA Nmir_018. The lower part shows the microarray slide (left) with a positive signal for Nmir_018 (white frame), and an agarose gel electrophoresis (right) of the loop-stem RT-PCR fragment for Nmir_018 (white frame).

To investigate how many of the ml-ncRNAs in the FANTOM3 might actually be me-ncRNAs, we developed the software PriMir to predict pre-miRNA sites within the reported 34,030 ml-ncRNA population (Figure 1). PriMir first establishes a score matrix (PriMir Score matrix, PMS matrix) to identify the stem-loops with the highest probability of being actual pre-miRNAs. The score matrix is established on the basis of 11 sequence and secondary structure characteristics of stem-loops in the training and background sets (see *Methods *for details). Because hairpins in the training set often contain flanking sequences around the precise pre-miRNAs, we annotated the exact position of the pre-miRNA in each hairpin in the training set according to that of the corresponding miRNA [8,9,20]. For short hairpins in miRBase we ran a Blastn search on the genomic sequence to obtain the necessary 10 nt flanking sequences. In order to create a hairpin background set representing the distribution of 11 features of stem-loop structure sequences with random length, we randomly reduced the lengths of the hairpins identified in the ml-ncRNAs. For each entry in the matrix, we calculated the ratio between the frequencies of each feature value in the training and background sets, and added it to the PMS matrix. All entries in the matrix were calculated according to the definition (A_ij_). For a given feature i with the value j, f_i_(j) and h_i_(j) are the frequencies of this feature having the value j in the training and background sets, respectively. X_i _is the feature value set of feature i. If a given value j belongs to X_i, _A_ij _is defined as the log 2 value of f_i_(j)/h_i_(j). Otherwise, A_ij _was assigned the minimum value of ***A*ixi**.

The next step was to predict the possible miRNA-encoding ml-ncRNAs. PriMir extracted about 184,000 hairpins (length >= 45 and paired bases >= 18) from the 34,030 ml-ncRNAs. To pick out the most likely pre-miRNA candidates we analyzed the conservation rate between mouse and rat for these sequences. In order to establish a threshold for the conservation filter, we aligned the 220 known mouse pre-miRNAs in the training set to the rat genome using Blastn. This resulted in 160 pre-miRNA sequences (> 70%) complying with two criteria: 1) The alignment lengths were larger than 45 nt, and 2) the identity of the alignment was 98% or higher. Therefore, we used these criteria for PriMir filtration, and obtained 4463 non-redundant conserved hairpins between mouse and rat, including 18 hairpins containing known pre-miRNAs.

Next, we used PriMir to predict pre-miRNA candidates from these 4463 hairpins based on the PMS matrix. For each conserved hairpin, PriMir predicts a potential pre-miRNA candidate and calculates a PriMir score based on the PMS matrix. The PriMir score value S is defined as the sum of the scores of all features for a given hairpin:

$$S=\sum_{i=1..11}Aixi$$

Here x_i _is the value of feature i.

To reduce the number of false positives, PriMir score "7" was used as a cutoff value. This is a stringent criterion, as ROC curve analysis (see *Methods *for details) of the PriMir performance showed that the AUC (area under curve) is approximately 0.99, and that the false positive rate is 0 at a PriMir scores of 7 (see Figure S4A in Additional file 1). We identified 84 pre-miRNA candidates with PriMir scores of 7 or higher, corresponding to 97 potential me-ncRNAs. Among these me-ncRNA candidates, 17 were included in set of 22 known me-ncRNAs; thus, the remaining 80 represent novel me-ncRNA candidates and altogether 102 me-ncNRAs were picked out finally.

To further evaluate the performance of the PriMir prediction software, we carried out cross-validation analysis (see Performance analysis in the *Methods *part), which gave AUC values between 0.971 and 0.984, suggesting the prediction results are reliable. (see Figure S4B in Additional file 1). During the course of this work, there were published three miRNA prediction algorithms [21-23] that were available for use on a local computer. A comparison between PriMir and these three algorithms suggested that the PriMir method is at least equal to and may in some respect outperform these three methods(see Figure S4A in Additional file 1). Furthermore, in order to get an estimate of which of the 11 stem-loop features contributed most to the identification of the pre-miRNAs, we carried out a simplified analysis of this problem by investigating the effect of each feature when running PriMir on the positive and negative test sets (see Performance analysis in the *Methods *part). This identified five features with an apparently contribution: the number of paired bases in the 10-bp up- and down- stream extensions of the pre-miRNA; the total bulge size of the pre-miRNA; basepairs in the pre-miRNAs; basepairs in the mature miRNA and the minimum free energy of the pre-miRNA(see Figure S5 in Additional file 1).

### Experimental validation of the predicted miRNAs

To experimentally validate the expression of the miRNAs encoded by the predicted me-ncRNA we spotted a microarray [24] with 168 26-nt probes corresponding to both arms of the 84 predicted pre-miRNAs, and hybridized this to size-fractioned RNA extracted from mouse tissues obtained from different developmental stages (see *Methods*). The microarray gave positive signals for 46 probes (see Figure S1 in Additional file 1), corresponding to 40 different pre-miRNAs. Of the 46 miRNAs, 14 had already been registered in miRBase 8.0, whereas the remaining 32 miRNAs, corresponding to 30 me-ncRNA candidates, are novel discoveries. (During the course of our work, 5 of the 32 novel miRNAs were also reported in the recent miRBase 9.2. release, thus lending further support to validity of our predictions.) As an additional validation we carried out stem-loop RT-PCR [25] (followed by sequencing) of the 32 novel miRNAs detected by the microarray, obtaining positive results for 26 of them (see Figure 1C, and Figure S2 in Additional file 1).

The expression levels of the investigated miRNAs appeared to be very low (Northern data, not shown). To make a comparison to the corresponding me-ncRNA expression levels we downloaded expression data for 20 different tissues for 15 of the experimentally supported me-ncRNAs from the Riken Expression Array Database (READ) [26]. The analyses showed that the average expression levels of the me-ncRNAs were similar to those of the entire ml-ncRNA set, and that a few of the me-ncRNAs are relatively high expression levels in a limited number of tissues (*P*-value < 0.02), such as transcript AK132542 (Accession number in DDBJ) in pancreas, AK008483 in thymus and skin at neonate day 10, AK136882 in liver and pancreas. Thus, there appear to no strong correlation between the expression levels of the me-ncRNAs and their encoded miRNAs.

Together with the 22 me-ncRNAs corresponding to known miRNAs, we altogether obtained a set of 52 experimentally supported me-ncRNAs. Given that the miRNAs may be tissue or cell type specific, and/or only be expressed during a limited time interval or under specific physiological or environmental conditions, we regard the rest 50 as yet unsupported me-ncRNA candidates. See Table S2 in Additional file 1 for more information on all the 102 me-ncRNAs and candidates.

### Motifs of the me-ncRNAs

**Figure 2:**
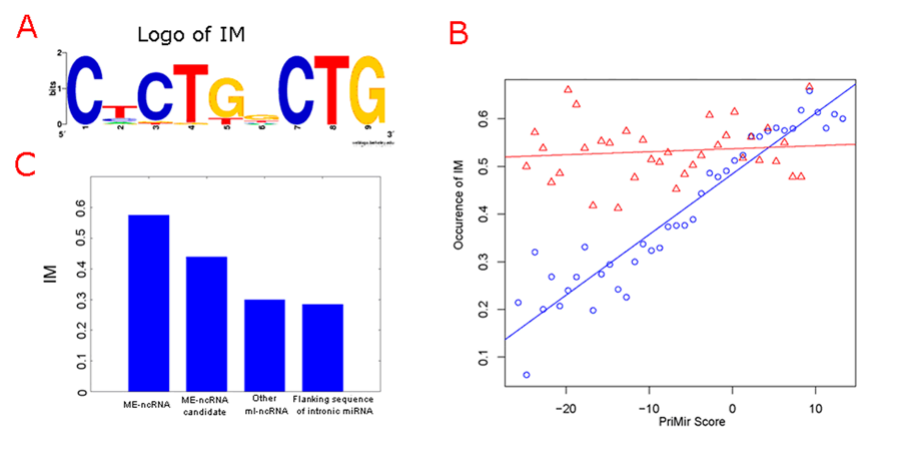
**The internal motif (IM) of the me-ncRNAs**. A: Logo of the IM (WebLogo [52]). B: The relationship between IM frequency and PriMir Score. The ml-ncRNAs were binned according to their PriMir score, and the fraction of transcripts with the IM were plotted. Blue circles: All ml-ncRNAs. Red triangles: ml-ncRNAs with conserved stem-loops. C: Frequency of IM in experimentally supported me-ncRNAs, unsupported ME-ncRNA candidates, other ml-ncRNAs, and in flanking sequence of intronic miRNAs.

Sequence analysis of the 52 experimentally supported me-ncRNAs revealed an internal motif (IM) with the consensus sequence CNCTGNCTG (Figure 2A, Table 1), which was clearly more frequent in the me-ncRNAs than in other analyzed sequences (Figure 2C). To test whether this motif is also a feature of other miRNA-encoding transcripts, we also searched for the motif in the vicinity of intron-encoded miRNAs. Although the motif do occur (28%) in this context, it is far less frequent than in the me-ncRNAs, possibly suggesting that this motif is a characteristic of miRNAs processed from the exonic parts of their primary transcripts. For the entire ml-ncRNA set we also found a very strong correlation between occurrence of IM and the highest PriMir score of an ml-ncRNA transcript (R^2 ^= 0.9, *P*-value = 2.2 × 10^-16^; Figure 2B); that is, the likelihood of an ml-ncRNA having an IM sequence is related to the likelihood (as given by the PriMir score) of the ml-ncRNA encoding an miRNA. Quite tellingly, for the set of 3,670 ml-ncRNAs with conserved stem-loops the correlation between PriMir score and occurrence of IM is very low (R^2 ^= 0.01, *P*-value = 0.5; Figure 2B), most reasonably because an ml-ncRNA with a conserved stem-loop has a high likelihood of encoding a miRNA, and therefore also of containing an IM sequence, irrespective of the typicality of its stem-loop characteristics (i.e. its PriMir score).

**Table 1:** Positional Weight Matrix for the IM sequence

**Position**	**A**	**C**	**G**	**T**	**Consensus**
1	0	1	0	0	C
2	0.07	0.18	0.07	0.67	T
3	0	0.92	0.04	0.04	C
4	0	0	0.04	0.96	T
5	0	0	0.85	0.14	G
6	0.07	0.14	0.55	0.22	G
7	0	1	0	0	C
8	0	0	0	1	T
9	0	0	1	0	G

### Structure and conservation of me-ncRNA loci

For analysis of their conservation and gene structure, the sequences of the 34,030 ml-ncRNAs were mapped to the mouse genome (mm7, see *Methods*). The gene structure of the me-ncRNAs is generally more complex, with 44% of the transcripts being spliced, compared to 29% for the entire ml-ncRNAs set. In order to evaluate the conservation of the me-ncRNAs and ml-ncRNAs, we assigned PhastCons [27] scores based on 17 vertebrate genomes to all base pairs in their corresponding genomic sequences, and average PhastCons scores (APCSs) were calculated as a measurement of conservation level (Table 2). In accordance with previous research [28], we found that both overall and stem-loop sequence conservation is weak (~23%) for the ml-ncRNA set. In contrast, the overall sequence conservation for me-ncRNA is relatively high (37%), and for the pre-miRNA hairpins the level of sequence conservation is striking (81%).

**Table 2:** Splicing and conservation characteristics of the me-ncRNAs

**ncRNA category**	**PUT**	**PST**	**ANEST**	CPMH*	CFLS*
ES me-ncRNAs	56%	44%	2.9	81	37
Other ml-ncRNA	71%	29%	3.7	23^#^	23

ES: Experimentally Supported; PUT: Percentage of Unspliced Transcripts; PST: Percentage of Spliced Transcripts; ANEST: Average Number of Exons of Spliced Transcripts; CPMH: Conservation of Pre-miRNA Hairpins; CFLS: Conservation of Full Length Sequence;

*Conservation was estimated as the average PhastCons score (APCSs), For "Pre-miRNA hairpins in other ml-ncRNAs", conservation is calculated for all PriMir extracted hairpins

The above analysis of the conservation of the me-ncRNAs was based on the genomic sequence conservation of 17 vertebrates, most of which are evolutionally distant to the mouse. The conservation characteristics of the me-ncRNAs between mouse and human were also analyzed. The me-ncRNAs were aligned to the human genome using BLAT and defined as conserved between mouse and human if the coverage was more than 50% and the identity more than 90%. Similarly, the pre-miRNAs were aligned to the human genome using Blastn and defined as conserved if the coverage was more than 80% and the identity more than 90% (Table 3). Direct sequence analyses between mouse and human only found that only 8 of the experimentally supported me-ncRNA were conserved, and for a considerable fraction of the rest (35%) not even their miRNA-encoding stem-loop structures were conserved beyond the rodents; thus, me-ncRNAs may for the most part encode species-specific miRNAs in mammals.

**Table 3:** Conservation of me-ncRNAs and their corresponding pre-microRNAs between human and mouse.

**ME-ncRNA**	**Corresponding pre-miRNA**	**Number of ES me-ncRNA/pre-miRNA**	**Number of me-ncRNA/pre-miRNA**
Non-conserved	Conserved	26/25	38/37
Non-conserved	Non-conserved	16/12	47/35
Non-conserved	Mixed*	2/4	2/4
Conserved	Conserved	7/6	13/12
Conserved	Non-conserved	1/1	2/2

ES: Experimentally Supported;

*Mixed: indicates that the same me-ncRNA contains both conserved and non-conserved pre-miRNAs

### Estimated numbers of me-ncRNAs

**Figure 3:**
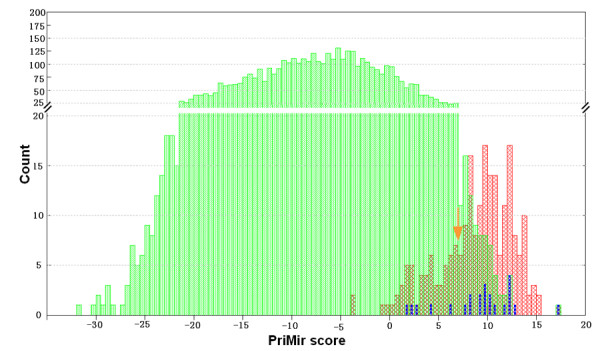
**The distribution of PriMir scores of the training set and the potential pre-miRNA candidates**. Red columns: the 220 known pre-miRNAs in the training set; blue columns: the 23 known pre-miRNAs in ml-ncRNAs; green columns: the predicted 4463 conserved hairpins. The red arrow indicates the cutoff value "7" PirMir used to predict candidate pre-miRNAs. Hundred and sixty-one (73%) of the 220 known pre-miRNAs fall above this cutoff. Of the 23 known pre-miRNAs located in ml-ncRNAs, 18 (78%) have scores higher than 7.

The above results as well as investigations in *Arabidopsis thaliana *[29] indicate that ml-ncRNAs encoding miRNAs may be a widespread phenomenon among eukaryotes. It is therefore of interest to get an idea of what fraction of the ml-ncRNA transcriptional output might actually be me-ncRNAs. The 97 me-ncRNA candidates reported above were identified using very stringent criteria, and both the number of conserved stem-loop structures and the presence of the IM would suggest that there may be a considerable number of me-ncRNAs in the ml-ncRNA population. To obtain an estimate of this number we built a simple model based on conservation of stem-loop sequences, PriMir score and the PriMir ROC curve. Among the 4,463 most conserved stem-loop hairpins (found within 3,670 ml-ncRNAs), there was 84 transcripts with a PriMir Score of 7 or higher. According to the ROC curve (see Figure S4A in Additional file 1), this score indicates a specificity of 100% and a sensitivity of 0.560, suggesting that there would be around 150 real pre-miRNAs. Considering further that only around 50% of pre-miRNAs fulfill our stringent conservation requirement, this results in more than 300 pre-miRNAs, corresponding to a slightly lower number ml-ncRNAs. Relaxing the PriMir cutoff value from 7 to 0 (since about 95% of the known pre-miRNA have a PriMir score of more than 0; see Figure 3), we obtain 738 stem-loops. A PriMir score of 0 corresponds to a specificity of 0.99 and a sensitivity of 0.893, which would indicate about 800 ml-ncRNAs encoding a miRNA. Thus, the number of me-ncRNAs in the mouse could vary from a lower estimate of around 300 up to 800 transcripts.

## Discussion and conclusion

Based on hairpin conservation and a comprehensive list of pre-miRNA features, we have designed a computational procedure which detected 80 novel me-ncRNA candidates in the mouse genome and provided experimental support for the expression of a substantial fraction of their encoded miRNAs. Through the above analyses we have shown that the me-ncRNAs differ from other ml-ncRNAs in gene structure and sequence conservation, and that their sequence and expressional characteristics are also different from other pri-miRNAs.

### The correlation between the internal motif and the PriMir score

An intriguing aspect of the analysis was the observed correlations between the presence of typical pre-miRNA characteristics (as represented by the PriMir score; PMS) and the occurrence of the internal motif IM within an mRNA-like ncRNA sequence. For the entire mRNA-like ncRNA collection there was a very strong correlation between the IM frequency and PMS, however, in the set of mRNA-like ncRNAs selected for hairpin sequence conservation this correlation was far weaker, despite the frequency of IM being higher in this set than in the entire mRNA-like ncRNA collection. There could be several explanations that would account for this discrepancy. The most straightforward is that the IM is associated with the miRNA encoding function of an ml-ncRNA, and that the processing of a stem-loop hairpin depends on either its interaction with general pri- and pre-miRNA processing factors (as indicated by its PMS value), or on more specific factors (in the case of conserved hairpins). In the first case, the IM would primarily be found associated with hairpins with high PMS values, where in the latter case, conserved hairpins should have a relatively high frequency of IMs, irrespective of PMS value. As the majority of IM-associated hairpins are not well conserved, this might imply that heavy reliance on sequence conservation may not be a particularly useful strategy for detection of a larger subset of me-ncRNAs. The strong correlation between IM and the PM score (which is likely to exemplify the typical pre-miRNA) in the full mRNA-like ncRNA collection therefore invites further work on computational miRNA detection based on other sources than sequence conservation. However, the IM sequence is quite short (containing only 7 partially conserved nucleotides), and further analysis of me-ncRNA sequences may reveal additional elements which could increase its predictive value.

### Biogenesis and function of the me-ncRNAs

Previous knowledge on miRNA biogenesis assumes that pri-miRNAs are processed into pre-miRNAs in the nucleus by the Drosha complex, and then transported to the cytoplasm where further processing by Dicer occurs, resulting in the mature miRNA [10]. The question of the sub-cellular localization of me-ncRNAs has not yet been investigated, but a few primary miRNA transcripts have been reported to accumulate in cytoplasm [30,31], The fact that me-ncRNAs are sufficiently stable to be cloned as full-length cDNAs, and that they retain several mRNA-like characteristics (splicing, capping, polyadenylation) would suggest that they may follow the path of coding mRNAs and be exported to the cytoplasm. Increasing evidence that post-transcriptional miRNA processing is subject to regulatory activity [31-34] and the apparent differences in the expression levels of the me-ncRNAs and their encoded miRNAs found here, further allows for a hypothesis in which me-ncRNAs constitute a miRNA storage form, possibly in addition to other functional properties of the intact me-ncRNA transcript. This storage may be maintained through low transcriptional and degradation activity of the me-ncRNAs, and producing only low levels of mature miRNA release under normal conditions. Upon some triggering event it could then enable a quick release of a larger amount of the mature miRNA through me-ncRNA processing without requiring transcriptional activation of the me-ncRNA locus. This in turn begs the question of whether there might exist a cytoplasmic pathway for miRNA maturation, or if the mature me-ncRNA re-enters the nucleus for processing by Drosha before the miRNA is released. In any case, there is the possibility that me-ncRNAs may have other cellular functions in addition to that of encoding miRNAs, as found for a number of other ml-ncRNAs [35-37], and that they therefore exist in other cellular compartments and are maintained at higher steady state levels than pri-miRNAs whose only role is to generate mature miRNAs.

In fact, the phenomenon of long primary transcripts encoding shorter functional ncRNAs is by not limited to ml-ncRNAs encoding miRNAs. Whole-genome tiling array scans have revealed that many small RNAs have genomic loci that overlap with longer transcripts, and the longer RNAs may represent primary transcripts for the shorter mature RNAs [18]. It is thus not implausible that a fraction of the ml-ncRNAs may serve as vectors or storage forms for short ncRNAs, which are then released when needed to perform their cellular functions. Our finding that a considerable number of ml-ncRNAs actually encodes miRNA could suggest that serving as the primary transcript of various classes of short ncRNAs may be a common function of longer ncRNAs.

## Methods

### Databases and Software

Data collection: Sequences of 34,030 mouse ml-ncRNAs were downloaded from the FAMTOM3 database [38]. Known mouse miRNAs were downloaded from miRBase release 8.0 [39]. The mouse (mm7), rat (rn3) and human (hg17) genome sequences were downloaded from UCSC [40]. Expression profiles for ml-ncRNAs were collected from the Riken Expression Array Database [26,41].

PhastCons Scores: The conservation scores for alignments of 16 vertebrate genomes with mouse (PhastCons17Scores) were downloaded from the UCSC web site [42].

Sequence logos: Logos of sequences were generated by web server at UC Berkeley [43].

### Training and background sets

To create a training set we needed to elicit the common features of known pre-miRNAs. In the miRBase release 8.0, there are altogether 270 different hairpins corresponding to 301 mouse miRNAs. First, the pre-miRNAs containing shorter mature microRNAs (<20 nt) or whose mature microRNA sequence extended into the loop region of the predicted stem-loop structure were filtered out. From this set we then removed the pre-microRNAs whose stem-loop structures could not be predicted by RNAfold (using the pre-miRNA and 200 nt flanking sequence in both directions), This left 220 of the 270 hairpins to be used as the training set.

We also needed to construct a background set of non-pre-miRNA hairpins to estimate the background noise. We predicted RNA secondary structures of the 34,030 ml-ncRNAs in FANTOM3 using RNAfold [44,45], and extracted hairpins from them based on two conditions: 1) The length of the hairpin should be longer than 45 nt, and 2) the number of paired bases in the hairpin should be more than 28 (14 base pairs). This step resulted in about 184,000 predicted hairpins. To create a hairpin background set representing the distribution of 11 features of stem-loop structure sequences with random length, we randomly reduced the lengths of the hairpins and used all of them as the background set.

### The eleven features used by PriMir

PriMir predicts pre-miRNAs according to the PMS matrix, which is based on eleven features found in the sequence or secondary structure of known pre-miRNAs [46,47]. The eleven features are: 1) the total number of paired bases in the 10-bp up- and down-stream extensions of the pre-miRNA; 2) the total bulge size of the pre-miRNA, i.e. the total number of nucleotides in all bulges in the pre-miRNA; 3) the total number of paired bases in the pre-miRNA; 4) the length of the loop in the pre-miRNA; 5) the distance between the mature miRNA and the terminal loop; 6) the sequence bias of the first five bases in the mature miRNA; 7) the total number of paired bases in the mature miRNA portion of the pre-miRNA; 8) the minimum free energy (mfe) of the pre-miRNA stem-loop calculated with the RNAfold program; 9) the length of the pre-miRNA; 10) the GC content of the pre-miRNA; and 11) the GC content of the mature miRNA; (see Figure S3 in Additional file 1).

### Performance analysis

The reliability of the PriMir prediction method was evaluated by cross-validation analysis. The training and background sets used to establish the PMS Matrix was divided into five equal parts. Four of these parts were selected to establish the PMS Matrix, whereas the remaining one part (from both training and background set) was used to test the performance of PriMir method by using the ROC-curve analysis. The above analysis was repeated 5 times, each time using a different portion of the data as test data set (see Figure S4B in Additional file 1).

To evaluate the performance of PriMir with a ROC curve, we constructed a positive and a negative set of stem-loop hairpins. For the positive set, we aligned the 432 mouse pre-miRNAs in miRBase 10.1 to the rat genome (blastn; identity >= 98%, alignment >= 45 nt) and obtained 208 conserved pre-miRNAs. To obtain a fair appraisal of the PriMir method relative to other methods, we removed those pre-miRNAs that were included in the PriMir training set from the 208 pre-miRNAs, which left us with a positive set of 75 pre-miRNAs. To obtain a negative set, we downloaded 198,536 refseq exons from the mouse mm9 genome (UCSC genome browser) and predicted stem-loop hairpin (length >= 45, paired bases >= 28) with RNAfold. This gave 48,314 hairpins, which were aligned to the rat genome (blastn; identity >= 98%, alignment >= 45 nt). This yielded about 9000 conserved hairpins from which we randomly selected 500 to constitute the negative set.

### The me-ncRNA internal motif

We constructed an IM Position Weight Matrix (PWM) according to the MEME [48] analysis of 30 me-ncRNAs confirmed by stem-loop RT-PCR. Then all 52 experimentally confirmed me-ncRNAs were iteratively analyzed and the IM PWM optimized according to each new result. Table 1 shows the final constringent IM PWM after 4 rounds of iterative analysis. PWM score 7.0 was used in this study as the cutoff value for the occurrence of IM.

### Genome Mapping

The sequences of the 34,030 ml-ncRNAs were mapped to the mouse genome (mm7) through the following steps. Firstly, the sequences were aligned to the genome using BLAT with options -fine -q rna. Then, the coverage (number of matches/full length of transcript sequence) of each alignment was calculated, and the low-quality alignments with coverage of less than 70% were removed. Finally, the alignments were modified according to the positions of exons from neighboring alignments.

We mapped the sequences of hairpins instead of miRNAs to the mouse genome (mm7) using Blastn program. The alignment results with an alignment length equal to the length of the hairpin and an identity of 100% were extracted. Blastn was downloaded from NCBI [49].

### Tissues and total RNA extraction

0-day neonate C57 BL/6 mice were provided by Vitalriver Laboratory Animal Technology Co., Ltd., Beijing. 15-day embryonic C57 BL/6 mice were provided by the Department of Laboratory Animals of Peking University Health Science Center. Male adult mice were provided by the Laboratory Animal Center, Institute of Genetics, Chinese Academy of Sciences. Total RNAs were extracted from (1) brain and thymus of 0-day neonate C57 BL/6 mice, (2) brain of male adult C57 BL/6 mice, and (3) whole body of 15-day embryonic C57 BL/6 mice, using the Trizol reagent (Invitrogen, Carlsbad, CA, USA) according to the manufacturer's instructions.

### Detection of miRNAs by microarray

This work was carried out at CapitalBio Corp. in Beijing, China, according to their in-house technology for miRNA detection [24]. We designed 168 26-nt oligonucleotide probes corresponding to both arms of the 84 predicted pre-miRNAs. In addition, we designed 8 19–24 nt oligonucleotides possessing no homology with any known RNA sequence and produced 7 complementary oligos to simulate miRNAs by in vitro transcription. To facilitate subsequent hybridization, poly-Ts were added to the 5'-end of the probes, resulting in 42-nt oligonucleotide probes (see Table S3 in Additional file 1). Each probe was printed in triplicate using a SmartArray™ microarrayer. Low-molecular-weight RNAs (<200 nt) were isolated from total RNAs by the PEG precipitation approach [50], and labeled using T4 RNA ligase [33] Hybridization was performed using LifterSlip™. Arrays were scanned with a confocal LuxScan™ scanner and Data were extracted from the TIFF images using LuxScan™ 3.0 software.

### Detection of miRNAs by stem-loop RT-PCR

Stem-loop RT-PCR experiments were performed to validate the miRNAs detected by microarrays. The procedure was essentially carried out as described by Chen *et al. *[25] and all primers were listed in Table S4 (see Additional file 1). Briefly, small RNAs extracted from a mixture of total RNAs (1), (2) and (3) of C57 BL/6 mice (see "Tissues and total RNA extraction" above) using the *mir*Vana™. Then, PCRs were performed using 1 μl of the RT products as template in a 20 μl reaction volume with Taq DNA polymerase (Invitrogen, Brazil, Cat #10966-030). The reactions were incubated at 94°C for 5 min, followed by 40 cycles of 94°C for 15 sec, 45°C for 30 sec and 60°C for 30 sec, with a final incubation at 60°C for 2 min. The elongated PCR products (about 60 bp in size) were cloned into pGEM-T (Promega A3600) and sequenced at Invitrogen.

## Authors' contributions

SH, CL, YZ and RC conceived and designed the study. SH and CL performed the prediction method. HS, HH and DH performed the experimental work. SH, CL, GS, XZ and TL analysed the data. SH, HS, CL, GS, YZ and RC wrote the paper. All authors read and approved the final manuscript.

## Supplementary Material

Additional file 1 **Supplementary figures and tables**. Figure S1: Detection of miRNAs with microarrays. Figure S2: Detection of miRNAs with stem-loop RT-PCR. Figure S3: Illustration of the 11 features used by PriMir. Figure S4: The Performance of PriMir. Figure S5: The score distribution of the five features that gave the strongest contribution pre-miRNA stem-loop identification. Table S1: 22 ml-ncRNAs encoding known miRNAs. Table S2: Information on all 102 me-ncRNAs. Table S3: List of probes for microarray. Table S4: List of stem-loop RT-PCR primers Click here for file
