# Supplementary material for: MicroRNA-encoding long non-coding RNAs
Source: BMC Genomics. 2008 May 21;9:236. doi: 10.1186/1471-2164-9-236 (PMC2410135; doi:10.1186/1471-2164-9-236)
Supplement: Additional file 1 — Supplementary figures and tables. Figure S1: Detection of miRNAs with microarrays. Figure S2: Detection of miRNAs with stem-loop RT-PCR. Figure S3: Illustration of the 11 features used by PriMir. Figure S4: The Performance of PriMir. Figure S5: The score distribution of the five features that gave the strongest contribution pre-miRNA stem-loop identification. Table S1: 22 ml-ncRNAs encoding known miRNAs. Table S2: Information on all 102 me-ncRNAs. Table S3: List of probes for microarray. Table S4: List of stem-loop RT-PCR primers [file 1471-2164-9-236-S1.pdf]

Additional file 1

File format: PDF

Title: Supplementary figures and tables

Content:

Figure S1: Detection of miRNAs with microarrays.
Figure S2: Detection of miRNAs with stem-loop RT-PCR.
Figure S3: Illustration of the 11 features used by PriMir.
Figure S4: The Performance of PriMir.
Figure S5: The score distribution of the five features that gave the strongest contribution pre-miRNA stem-loop identification.
Table S1: 22 ml-ncRNAs encoding known miRNAs
Table S2: Information on all 102 me-ncRNAs.
Table S3: List of probes for microarray.
Table S4: List of stem-loop RT-PCR primers

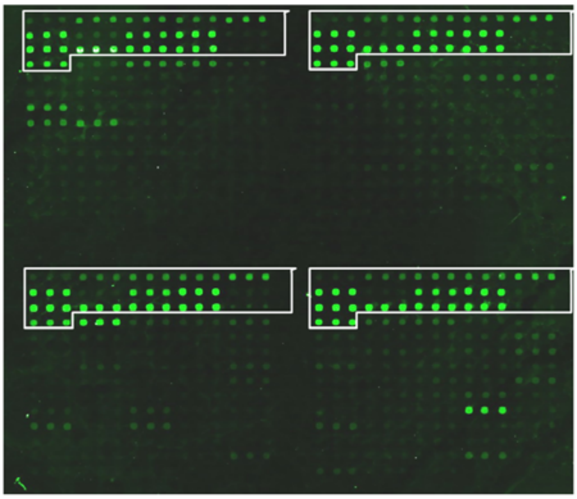

brain and thymus of 0-day neonate C57 BL/6 mice

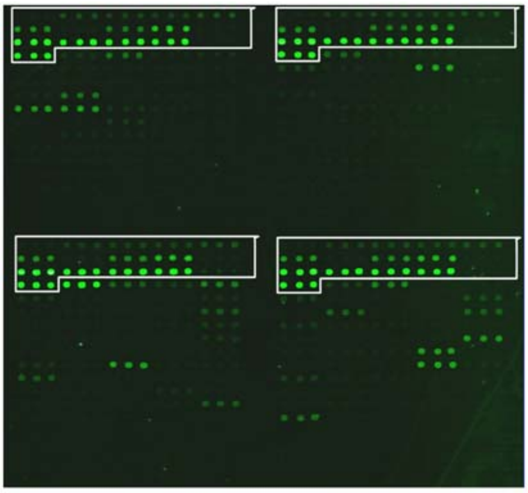

brain of male adult C57 BL/6 mice

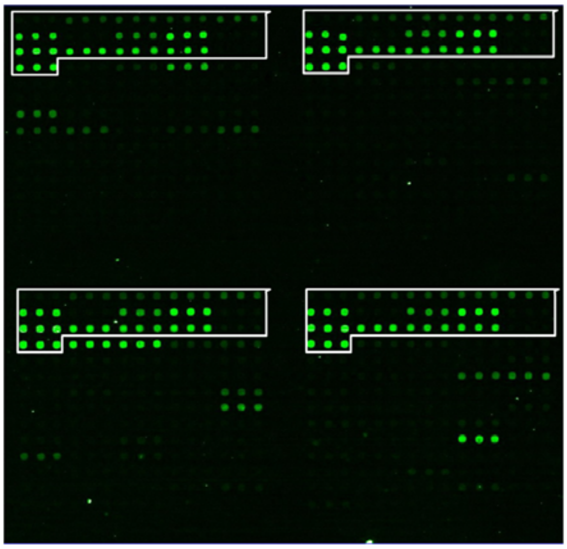

15 days old fetuses

Figure S1. Detection of miRNAs with microarrays. Low molecular weight RNAs (<200 nt) from (1) brain and thymus of 0-day neonate mice, (2) brain of adult male mice, and (3) 15 days old fetuses of C57 BL/6 mice were hybridized to microarrays with probes specific for the predicted miRNAs. White frames indicate control probes.

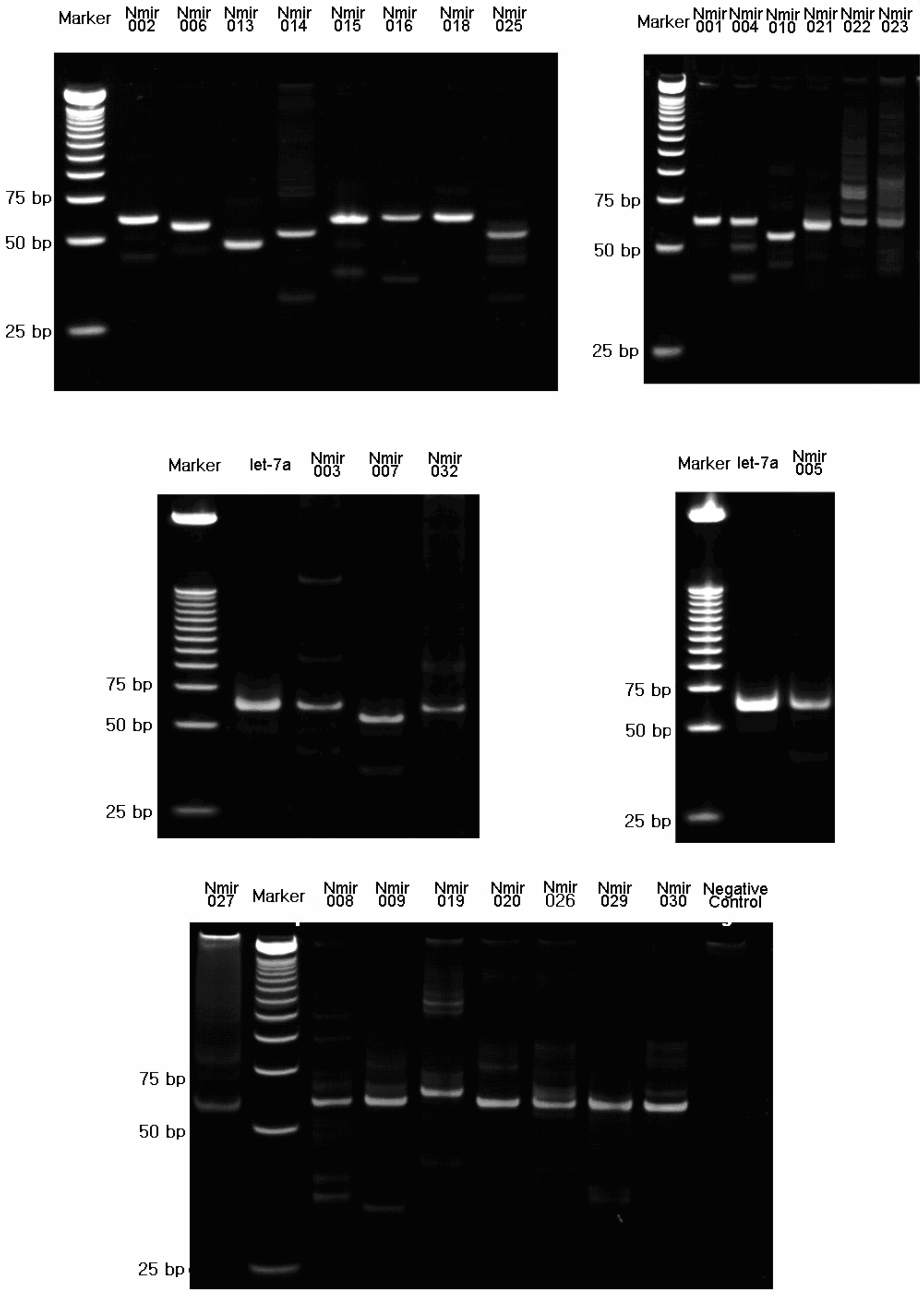

Figure S2. Detection of miRNAs with stem-loop RT-PCR. Low molecular weight (<200nt) RNAs were extracted from different tissues in 3 developmental stages: 1) brain and thymus of 0-day neonate C57 BL/6 mice, 2) brain of male adult C57 BL/6 mice, and 3) whole body of 15-day embryonic C57 BL/6 mice, and then mixed. 10 ng of the mixed RNAs were used as templates for RT reactions. Stem-loop RT-PCR of

let-7a was included as a positive control; and the same RT-PCR reaction as Nmir_002 was performed without reverse transcriptase as a negative control. 26 of the 32 miRNAs microarray detected were detected by stem-loop RT-PCR.

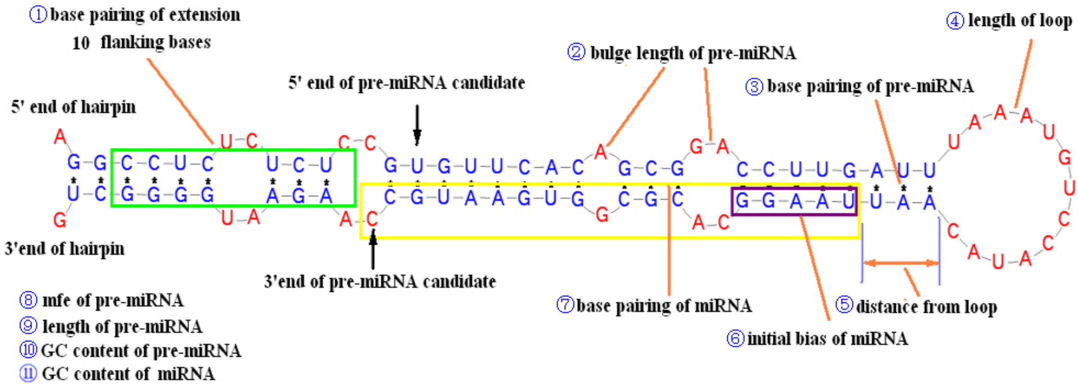

Figure S3. Illustration of the 11 features used by PriMir

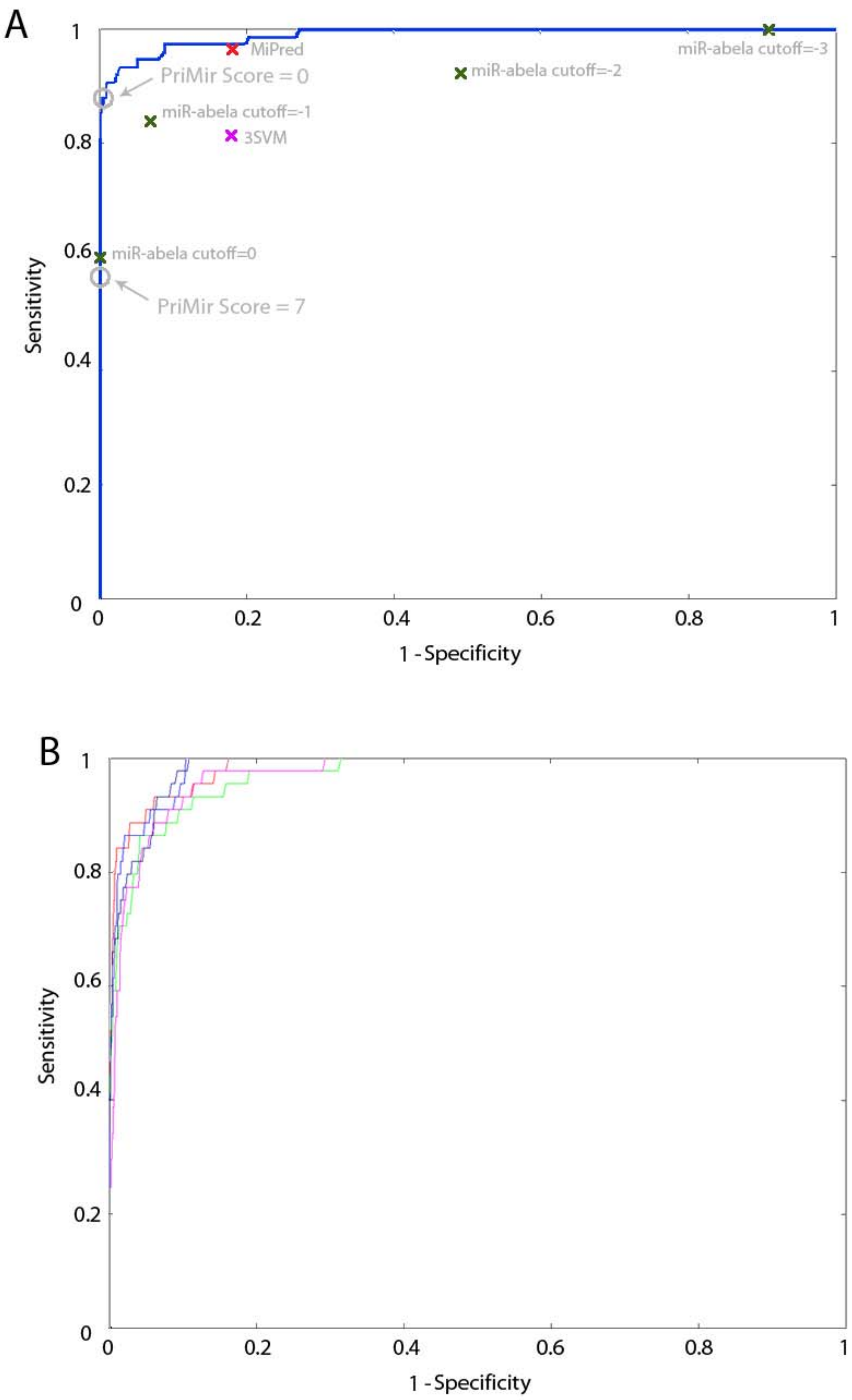
A
MiPred
PriMir Score = 0
miR-abela cutoff=-1
3SVM
miR-abela cutoff=-2
miR-abela cutoff=-3
miR-abela cutoff=0
PriMir Score = 7
Sensitivity
1 - Specificity
B
Sensitivity
1 - Specificity

Figure S4: The performance of PriMir. A. The PriMir ROC curve. The blue line represents the PriMir ROC curve. The Area Under Curve (AUC) is about 0.99. A PriMir score of 0 corresponds to a specificity of 0.99 and a sensitivity of 0.893, and the PriMir score of 7 corresponds to a specificity of 1 and a sensitivity of 0.56. The red crosses indicate sensitivity and 1-specificity for MiPred, the pink cross indicates the prediction results for 3SVM, and the green crosses indicates prediction results obtained with miR-abela with three different cutoff values. B. ROC-curves from the cross-validation of PriMir. The training and background sets were both divided evenly into five parts. Four parts were used for training of PriMir, and remaining set was used to test the trained PriMir. The five different curves represent the results of five independent tests, resulting in AUC values of 0.984, 0.971, 0.984, 0.972 and 0.983.

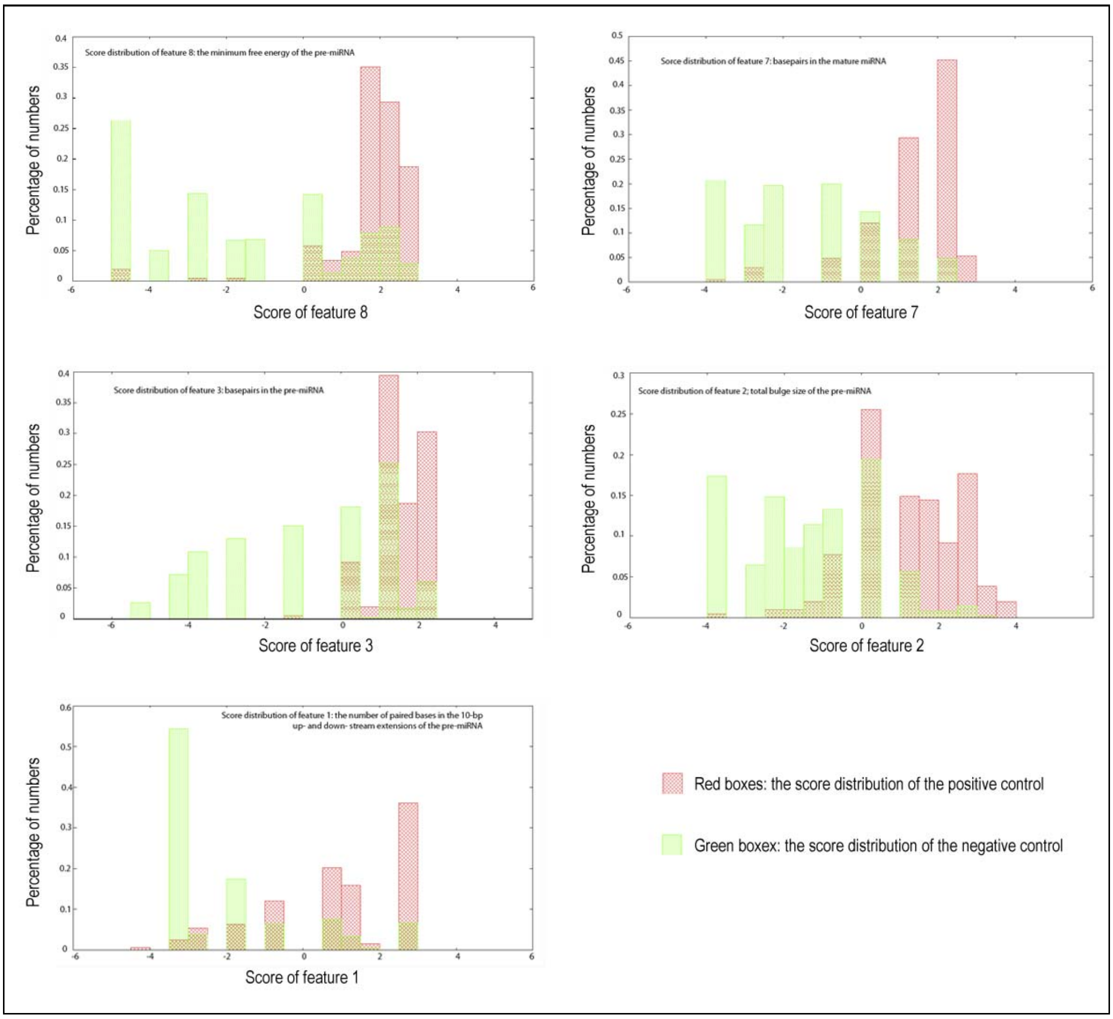

Figure S5: The score distribution of the five features that gave the strongest contribution pre-miRNA stem-loop identification.

Table S1. 22 ml-ncRNAs encoding known miRNAs

me-ncRNA ID	Clone Id in FANTOM3	miRNA
MENC_001	D630033A02	mmu-let-7i
MENC_002	A930011O12	mmu-mir-124a-1
MENC_003	8030499P14	mmu-mir-130a
MENC_004	F930027K19	mmu-mir-144
		mmu-mir-451
MENC_005	4833445I07	mmu-mir-191
		mmu-mir-425
MENC_006	G630041I22	mmu-mir-202
MENC_007	4631405K08	mmu-mir-205
MENC_008	4831414J02	mmu-mir-133b
		mmu-mir-206
MENC_009	6720427I07	mmu-let-7d
MENC_010	C030026K10	mmu-mir-22
MENC_011	9130016C20	
MENC_012	2210403K04	
MENC_013	5930426H09	mmu-mir-331
MENC_014	D230012O04	mmu-mir-214
MENC_015	9830169G21	mmu-mir-223
MENC_016	5330411G14	mmu-mir-377
MENC_017	B230304K20	mmu-mir-378
MENC_018	D930047H10	mmu-mir-138-2
MENC_019	B130046N22	mmu-mir-100
MENC_020	E130002N09	mmu-mir-181b-1
MENC_021	2010103J01	mmu-mir-215
MENC_022	6430411K18	mmu-mir-433

Table S2: Information on all 102 me-ncRNAs.

The table includes data on all me-ncRNAs corresponding to previously known miRNAs and experimentally verified miRNA, and data on me-ncRNA candidates without experimental support

ID of me-ncRNAs	Clone Id in FANTOM3	sequence of the contained pre-miRNA	miRNA sequence	name of miRNA[a]	Detected by microarray[b]	RT-PCR[b]	Conserved[c]	
							Me-ncRNA	Pre-miRNA
MENC_001	D630033A02	UGAGGUAGUAGUUUGUGCUGUUGGUCGGGUUGUGACAUUGCCCGCUGUGGAGAUAACUGCGCAAGCUACUGCCUUGCU	UGAGGUAGUAGUUUGUGCUGUUGGUC	mmu-let-7i (miRBase 8.0)	D	NA	NC	C
MENC_002	A930011O12	UGUUCACAGCGGACCUUGAUUUAAAUGUCCAUACAAUUAAGGCACGCGGUGAAUGCCAA	UAAGGCACGCGGUGAAUGCC	mmu-mir-124a-1 (miRBase 8.0)	D	NA	NC	C
MENC_003	8030499P14	GCUCUUUUCACAUUGUGCUACUGUCUAACGUGUACCGAGCAGUGCAAUGUUAAAAGGGCAU	CAGUGCAAUGUUAAAAGGGCAU	mmu-mir-130a (miRBase 8.0)	D	NA	NC	
MENC_004	F930027K19	GGGAUAUCAUCAUAUACUGUAAGUUUGUGAUGAGACACUACAGUAUAGAUGAUGUACUAG	GGGAUAUCAUCAUAUACUGUAA	Nmir_001	D	D	NC	C
			UACAGUAUAGAUGAUGUACUAG	mmu-mir-144 (miRBase 8.0)	D	NA		
		AAACCGUUACCAUUACUGAGUUUAGUAAUGGUAACGGUUCUC	AAACCGUUACCAUUACUGAGUU	mmu-mir-451 (miRBase 8.0)	ND	NA		C
MENC_005	4833445I07	CAACGGAAUCCCAAAAGCAGCUGUUGUCUCCAGAGCAUUCCAGCUGCACUUGGAUUUCGUUCCC	CAACGGAAUCCCAAAAGCAGCU	mmu-mir-191 (miRBase 8.0)	D	NA	C	C
		AUGACACGAUCACUCCCGUUGAGUGGGCACCCAAGAAGCCAUCGGGAAUGUCGUGUCCGCC	AUCGGGAAUGUCGUGUCCGCC	mmu-mir-425 (miRBase 8.0)	NA	NA		C
MENC_006	G630041I22	UCCUAUGCAUAUACUUCUUUGUGGAUCUGGUCUAAAGAGGUAUAGCGCAUGGGAAGA	AGAGGUAUAGCGCAUGGGAAGA	mmu-mir-202 (miRBase 8.0)	D	NA	NC	C
MENC_007	4631405K08	GUCCUUCAUUCCACCGGAGUCUGUCUUAUGCCAACCAGAUUUCAGUGGAGUGAAGCUCAG	UCCUUCAUUCCACCGGAGUCUG	mmu-mir-205 (miRBase 8.0)	D	NA	NC	C
MENC_008	4831414J02	GCUGGUCAAACGGAACCAAGUCCGUCUUCCUGAGAGGUUUGGUCCCCUUCAACCAGCUA	UUGGUCCCCUUCAACCAGCUA	mmu-mir-133b (miRBase 8.0)	D	NA	NC	C
		ACAUGCUUCUUUAUAUCCUCAUAGAUAUCUCAGCACUAUGGAAUGUAAGGAAGUGUGUGG	UGGAAUGUAAGGAAGUGUGUGG	mmu-mir-206 (miRBase 8.0)	ND	NA	NC	NC
MENC_009	6720427I07	AGAGGUAGUAGGUUGCAUAGUUUUAGGGCAGAGAUUUUGCCCACAAGGAGUUAACUAUACGACCUGCUGCCUUUCU	AGAGGUAGUAGGUUGCAUAGU	mmu-let-7d (miRBase 8.0)	D	NA	NC	C
MENC_010	C030026K10	AGUUCUUCAGUGGCAAGCUUUAUGUCCUGACCCAGCUAAAGCUGCCAGUUGAAGAACUGU	AAAGCUGCCAGUUGAAGAACUGU	mmu-mir-22 (miRBase 8.0)	D	NA	NC	C
MENC_011	9130016C20						NC	
MENC_012	2210403K04						NC	
MENC_013	5930426H09	UUCUAGGUAUGGUCCCAGGGAUCCCAGAUCAAACCAGGCCCCUGGGCCUAUCCUAGAACC	GCCCCUGGGCCUAUCCUAGAA	mmu-mir-331 (miRBase 8.0)	D	NA	NC	C
MENC_014	D230012O04	UGCCUGUCUACACUUGCUGUGCAGAACAUCCGCUCACCUGUACAGCAGGCACAGACAGGCAGU	UGCCUGUCUACACUUGCUGUGC	Nmir_002	D	D	NC	C

			ACAGCAGGCACAGACAGGCAG	mmu-mir-214 (miRBase 8.0)	D	NA			
MENC_015	9830169G21	UGUAUUUGACAAGCUGAGUUGGACACUCUGUGUGGUAGAGUGUCAGUUUGUCAAAUACCCC	UGUCAGUUUGUCAAAUACCCC	mmu-mir-223 (miRBase 8.0)	D	NA	NC	C
MENC_016	5330411G14	AGAGGUUGCCCUUGGUGAAUUCGCUUUAUUGAUGUUGAAUCACACAAAGGCAACUUUUGU	AUCACACAAAGGCAACUUUUGU	mmu-mir-377 (miRBase 8.0)	D	NA	NC	C
MENC_017	B230304K20	CUCCUGACUCCAGGUCCUGUGUGUUACCUCGAAAUAGCACUGGACUUGGAGUCAGAAGGC	ACUGGACUUGGAGUCAGAAGGCC	Nmir_003 (miRBase 9.2: mmu-mir-422b)	D	D	NC	C
			CUCCUGACUCCAGGUCCUGUGU	mmu-mir-378 (miRBase 8.0)	ND	NA		
MENC_018	D930047H10	AGCUGGUGUUGUGAAUCAGGCCGACGAGCAGCGCAUCCUCUUACCCGGCUAUUUCACGACACCAGGGUU	AGCUGGUGUUGUGAAUC	mmu-mir-138-2 (miRBase 8.0)	NA	NA	NC	C
MENC_019	B130046N22	AACCCGUAGAUCCGAACUUGUGCUGAUUCUGCACACAAGCUUGUGUCUAUAGGUAUG	AACCCGUAGAUCCGAACUUGUG	mmu-mir-100 (miRBase 8.0)	NA	NA	NC	NC
MENC_020	E130002N09	AACAUUCAUUGCUGUCGGUGGGUUGAACUGUGUAGAAAAGCUCACUGAACAAUGAAUGCAAC	AACAUUCAUUGCUGUCGGUGGG	mmu-mir-181b-1 (miRBase 8.0)	NA	NA	NC	C
MENC_021	2010103J01	AUGACCUAUGAUUUGACAGACCGUGCAGCUGUGUAUGUCUGUCAUUCUGUAGGCCAAUA	AUGACCUAUGAUUUGACAGAC	mmu-mir-215 (miRBase 8.0)	NA	NA	NC	NC
MENC_022	6430411K18	UACGGUGAGCCUGUCAUUAUUCAGAGAGGCUAGAUCCUCUGUGUUGAGAAGGAUCAUGAUGGGCUCCUCGGUGU	UACGGUGAGCCUGUCAUUAUUC	mmu-mir-433 (miRBase 8.0)	NA	NA	NC	C
MENC_023	A130074J23	CUGAGGGGCAGAGAGCGAGACUUUUCUAUUUUCCAAAAGCUCGGUCUGAGGCCCCUCAGUC	CUGAGGGGCAGAGAGCGAGACU	Nmir_004	D	D	NC	C
			AGCUCGGUCUGAGGCCCCUCAG	Nmir_005 (miRBase_9.2: mmu-mir-423)	D	D		
MENC_024	1110017L17	UCUACUCUUGACCAAAGUUUUGUAAGAAACAAUACAGAAUUUGGGGACGGGAGGGAGGAU	AUUUGGGGACGGGAGGGAGGAU	Nmir_006	D	D	NC	NC
MENC_025	9830134K06						NC	
MENC_026	2010209012	AGGAAGCCCUGGAGGGGCUGGAGGUGAUGGAUGUUUUCCUCCGGUUCUCAGGGCUCCACC	AGGAAGCCCUGGAGGGGCUGGAGG	Nmir_007 (miRBase_9.2: mmu-mir-671)	D	D	NC	C
MENC_027	2400007E14	GAGGUAUCUGCUGCGCCUGAGAUGAGUAAACUGUCGUUUCGGGCGCGGGCGCUGGACGCCUCG	GGCGCGGGCGCUGGACGCCUCG	Nmir_008	D	D	C	C
MENC_028	2610025H06	CUCUCCCCUACCACCUGCCUCUUCCCUGCUGUGGAGCAGGCAAGGGAGAGGGUGAAGGGAG	CUCUCCCCUACCACCUGCCUCU	Nmir_009	D	D	C	NC
			GCAAGGGAGAGGGUGAAGGGAG	Nmir_010	D	D		
MENC_029	2900029M20	GCGCCGGCAGCGCGGGGCCGUUGCCCAGUCUUUGCAGUUAGAGCCCCAUCUCUCUGGCGUUG	GCGCCGGCAGCGCGGGGCCGUU	Nmir_011	D	ND	C	C

MENC_030	6330521N15	UCGCCUUGUCGUACUUGUAGCUUUGCAUGGCGCUGGCUGCGGGGAGCCUCCCGGGGCGAUG	CGGGGAGCCUCCCGGGGCGAUG	Nmir_012	D	ND	NC	C
MENC_031	9330164G18	UUCCUCUUCUUCUUGGCCGGGUUGGCUCCGCCAGCUACCCCCGAGGAGGACGAGGAGGA	CCCCCGAGGAGGACGAGGAGGA	Nmir_013	D	D	NC	C
MENC_032	9330178L02	CACACUCAUUACAGUGAUGUCUUUUGGAACGACCAUGGACUCUCUGAUGGUGGGUGAGGAG	CUCUCUGAUGGUGGGUGAGGAG	Nmir_014	D	D	NC	NC
MENC_033	A330012E03	CUUUGGAUGGAGAAAGAGGGGGGAAGUGCGAGCUCCUUCAACUCGUUCUGUCCGGUGAG	CUUUGGAUGGAGAAAGAGGGGG	Nmir_015	D	D	NC	NC
			UCAACUCGUUCUGUCCGGUGAG	Nmir_016	D	D		
MENC_034	A430080L19	CGCGGGGGCCGGCUUUAUUUCCCGCCUUUGCGGUGGACUAGAAAGGAGUCACCCCCGGGA	CGCGGGGGCCGGCUUUAUUUCC	Nmir_017	D	ND	NC	NC
MENC_035	A530020N14	AGGUCAAGGUUCACAGGGGAUCAAGAAAUAUUAUUCUUGUAUAAUCUGUUUACUUUGACCUAA	AGGUCAAGGUUCACAGGGGAUC	Nmir_018	D	D	C	C
MENC_036	B130042C21						C	
MENC_037	C920030H05						C	
MENC_038	4931433A09	AGCGAUGGCCGAAUCUGCUUCCUCCUGGUUAGAAUGAAUGUGCUAAGUGGAUUUCAGAUUCUGCUCAUUCCGUUGA	AGCGAUGGCCGAAUCUGCUUCC	Nmir_019	D	D	NC	NC
MENC_039	D930019D21						NC	
MENC_040	B230208K21	AAGCUAGAAGAGGGCGGAGCCUGGAGGGGACACCCGGGCCGCCCUCUCUGGUCCUUCA	GGCCGCCCUCUCUGGUCCUUCA	Nmir_020	D	D	NC	NC
MENC_041	B830003K07	AUUCCCUGAGUGAACGAGUUGAGGCGGCCCCGACUGCCGCUCGUACUCCCGGGGGUCC	CCGCUCGUACUCCCGGGGGUCC	Nmir_021	D	D	C	C
MENC_042	C130030E18	GGAGUGUUUGCUGUAUAAUUGGACUUGAUAAAAUGUUUAGAGGUGCAGUAGGCAUGACUU	AGAGGUGCAGUAGGCAUGACUU	Nmir_022	D	D	C	C
MENC_043	C530029I03	CCUUCUUCUUCUUCCUGAGACAUGGCCCGGGCAGUGGCUCCUGGAAGAGGAACAAGUGUG	CCUUCUUCUUCUUCCUGAGACA	Nmir_023	D	D	NC	NC
MENC_044	C530039003						NC	
MENC_045	C920016N10	CCGCCGGGCCCUGCAAUAUGAAGGAGCAGCCCUCAUGUGCGGGCACCGGGCAUCCGAGCA	CCGCCGGGCCCUGCAAUAUGAA	Nmir_024	D	ND	NC	C
			GCGGGCACCGGGCAUCCGAGCA	Nmir_025	D	D		
		GUUCUGCUCCUCUGGAGGGAGGUUCCUGUUUGCACACCUCUCUUCAGGUAGAUUAAACAU	GUUCUGCUCCUCUGGAGGGAGG	Nmir_026	D	D		NC
MENC_046	E330019P22	GCUGCUGGAUGCGUUUGAUGGUCGCUCCCUUGGGGCCCACCACCAGUCCCACCACGCGGUAG	CACCAGUCCCACCACGCGGUAG	Nmir_027	D	D	NC	C
MENC_047	F730021I03	UUUCUCUGCUACCUGCCGGCAGGGCUUCCUCUCCCAGCCGCCAGCGGGCUGAAGAGGAGGG	GCCAGCGGGCUGAAGAGGAGGG	Nmir_028	D	ND	NC	NC
MENC_048	F830003A04	AGCAACCUCCUAGGGUUGUUGUGAGAAUUAAAUGAACUGCAGCAGCCUGAGGCAGGGCUG	UGCAGCAGCCUGAGGCAGGGCU	Nmir_029	D	D	NC	NC
MENC_049	F830206C10						NC	
MENC_050	F830211N05	CUCCCCCUCGCUGUCUCUUGCUGGCUGAUUUUAAUUCUCUCCUACCGGGAGCAGCAGAGGAUCUGGAGGU	GAGCAGCAGAGGAUCUGGAGGU	Nmir_030	D	D	NC	C
MENC_051	IOC0030C13	ACUGGUGCGGAAAGGGCCCACAGUGGACUUGGUACACUGUAUGCCCUAACCGCUCAGUCC	CUGUAUGCCCUAACCGCUCAGU	Nmir_031 (miRBase_9.2:	D	ND	NC	C

				mmu-mir-675-3p)				
MENC_052	M5C1091A08	CCCUCAGGCCACCAGAGCCCGGAUACCUCAGAAAUUCGGCUCUGGGUCUGUGGGGAG	GAAAUUCGGCUCUGGGUCUGUGGGGAG	Nmir_032 (miRBase_9.2: mmu-mir-760)	D	D	NC	C
MENC_053	A430024D24	AGGUACGGUUGCCACGUGAACACACAUGUGCUGUGAGCAUGUGGUUAGCGCCGUCCUCA			ND	NA	NC	NC
MENC_054	K430345014	GCCACCACUUCCUGAGAGCCGGGGAUACCCAUCCCGGACGUCACGGAGGAGUCGAGGCGGCGG			ND	NA	NC	C
MENC_055	A130028J20	CUUUUUCAUGAGAGUUGAGAAGGCCUGGCCUUGGCCUUUUGUUUCUUCAUGAGAAGGU			ND	NA	NC	C
MENC_056	A430106D13	GAAUGUGUGAUUGCUUUGCAUGACUGCAAUGGAGAUGUCAACAGAGCCAUCAAUGUUCUC			ND	NA	NC	C
MENC_057	A930005K04	UCGCCGAUCUGCCCCAGUUGGAGCGCGAAGUCCUCCAGGUGGGCGAGAUGAUCGACGACA			ND	NA	NC	NC
MENC_058	2610301N02	GGGACACCCUUUGAAGAUGGUAAGUAAUUCUUAUUAUCUACAUAUUAAAAGGGUGCCCUU			ND	NA	NC	NC
MENC_059	2610301G21				ND		NC	
MENC_060	5330400K14	GAGAGGUGUUGUCUUUUGCCAAGAAGGGCACUUGGUGUCUUGGCAGAAGGCAGAGGGCUGCCC			ND	NA	NC	NC
MENC_061	9930009J05	GAUCGGUACCCCUCUGGGAACUGUGGGCUCUAUUACAGCUCAGGCUGGUGGUUUGAUUCA			ND	NA	NC	NC
MENC_062	B130001P19	UUUCUGGCCAGUGAAGAGAUCUACAUUAACCAGCUGGAAGCUCUGUUACUGCCAGGCAUC			ND	NA	NC	NC
MENC_063	D430036I17	GUCUUGGACCAGAGGCGGCCAAGAUACCAGACUGGUGCUUGGAGCCGCCAGUGCCGGAUAG			ND	NA	NC	NC
MENC_064	D630021F02	GGAGAUCAGGUUUGCACACUGUCUUGAGAACAAUGUUCCAGUGAGGAAUCCUGAUCUCUCG			ND	NA	NC	NC
		UGCGGUGUGUGUGUCCCCACCACGGCACUGUUCCCUUGUGGUGGUCGGACUACAACCCAAC			ND	NA		NC
MENC_065	F930104J06	AGUGUGAGAAGACAAAAUGAGAUACGUUAUGAAAGGUAACUGUAUUGUUUUCUCGCUCUGC			ND	NA	NC	C
MENC_066	I920046F24	UGCCUGCAUCUGAUUGGUCUGUGGGGUAUAUUCUUGAUAGCUAAUUGAUGGAGGCGAG			ND	NA	NC	NC
MENC_067	A130050K09	CAUAGGCUCCUAUGCUUGAACUCUUGGUCUCUAGUUCAUGGCACUGUUUUGGGAGUCUGUGGG			ND	NA	NC	NC
MENC_068	6720435L14	GAGGAAGUCCGGCAGACUUGUGGCUACAUCUCUGGCCAGUUUGACUUGGGUGAGUUCUCGU			ND	NA	NC	C
MENC_069	4732419F12	CUCUGGCUGAAGCCUGGCUCCUACUCUGAGAGGAGUGGCCAGUGAUUUCAGCUGCAGCU			ND	NA	NC	NC
MENC_070	A830006M23	CCAGGAGCCCACCUCUGUCACUGAGCCUAGUGGCAGCCAGGUGUCACUUGCAAGCUUUGGGU			ND	NA	NC	NC
MENC_071	B230380C22	AGUGGCUGUUGCUAUUCUGGUAUCACUCCUCUACCCUGGGUAGAAACUAAGCAACAGCCCUUC			ND	NA	NC	NC
MENC_072	A230053F13	CAUUUCCUGCAGCCCAGUGGCCUGCUGUACCUUCAGGUACACAGGGUUAUGUAGGACGGG			ND	NA	NC	NC
MENC_073	1110014E10	GGUUCAGCUAUGGUGCUAAUCAAUAGGUAUUUCAGUGUCAGGUUCCAUAGCUGAAUGCCAU			ND	NA	C	C

MENC_074	2010208G19	UUUGGGGAACUGCUGGAUAUGCCUAAUGUUAGAGAGGUGUGUUGCUUGCCUGGGUAGUCCCCAAAGA			ND	NA	C	NC
MENC_075	2010013M14	UGCAUGUCUGUACUUUGCUGAGUUUCUGUGUUCCUGCAGCAGUGGUCAGAACAUGCUGA			ND	NA	NC	NC
MENC_076	D130042G11	AGGGGAGUUUUCUUUCAAAAGAAUGGCUUCCAUCCAUUUGAAGGAAACCCCUCCCCCAC			ND	NA	NC	C
MENC_077	3526402003	UGCCCCUGACCCCACUGCUGUUUGCUGUACAUAGAGGCUAAGUGGGGUGAAGUGGGCAGG			ND	NA	C	C
MENC_078	4.93E+20	UUAUCCGGUUCCCUUAAUCCUUUAGGUACUUACACGACGUUGGGUGAGGGGAGCUGGAAGAU			ND	NA	NC	C
MENC_079	4930573F14	UGAGCCACUUGGCCCAUUUUAAAGGCUACACUUGAUUAAAAUUGGGACCAAGUUCAUCAUG			ND	NA	NC	NC
MENC_080	5830407M18	CCAUGAGCUGCUCCGCCUACAGCCUGGUGCCUUCCAACUGCGCGGUGAGCAGCUCCUGGUG			ND	NA	NC	NC
MENC_081	6030458J16	GGGUCUGGGCUGGAAAAAUCCUGGUGUUUUGAAUAUGUUCAGGUCCAGUUCAGACUCUUG			ND	NA	NC	C
MENC_082	G530004A22						NC	
MENC_083	G530143B20						NC	
MENC_084	I530014C21						NC	
MENC_085	7420435F03	CGCCUGGGCCUCAUGGAAAUGAUCGCUUUCGCCAAGAUCUUCUGUAAGGGCCAGGUGUC			ND	NA	NC	NC
MENC_086	9330159N21	UUUCUUGGUCUGCCUGGCCUCUGUAAAUGUGUUGAAUGAUAGAGCUAUCAGGCAUGCUAAGGCAACA			ND	NA	NC	NC
MENC_087	9430042D23	UGUGUUUGAUGCCAAUAAUGGUUGCCAGUGGCACCGAGGUUGGCAUCAGACCCUCUUU			ND	NA	NC	C
MENC_088	9630058L18	UCACAUACAAAAGAUGGUGGAUCUUGUGGCCCAGAAGUCCCACUUCUUUGUGUGUGAGA			ND	NA	C	C
MENC_089	2610025H06							
MENC_090	6030473A16							
MENC_091	9.93E+25	GUGUGUGAUGUGUAUGUAGGCAAAUAUUUACAUAUUUAUACAUACACAGGUGCAUACAC			ND	NA	NC	C
MENC_092	A130012M23	AUGCUUGGGAAGGAGCCACCUGCUAUGGGAAAGGCAGUGGACUUCCUGUUCCCAGCAUAG			ND	NA	NC	NC
MENC_093	A330041B18	UUUUGUCUCCUCUGCAGUCUGACUGGUUCUUCAGUGAUGAUGAGGACAAAGGAGAGAGAGUA			ND	NA	NC	C
MENC_094	A430103D08	AAAAGGCUCACCUCCAUCACUACCUGCAAGUUGAUGGGAUGGAGGAAAGCACUUUUAC			ND	NA	NC	NC
MENC_095	A430080K14	GUUUGCUCAUUUCCCUUCGGAUUUAGGUAACGGUUAAAUUUGGAAGAGGUGGGCGAGCG			ND	NA	C	C
MENC_096	I730043G20						C	
MENC_097	A630093L23	UGCGAGGAUUGAAUUGGCUCAUCUCUCUGUAUGAGAAUGGCAUCAAUGGGAUCCUUGCAGA			ND	NA	C	C

MENC_098	D030040M08						C	
MENC_099	A730069D24	UCGGCAAACUUCCAAGAUACAGAGGACAUUCUUGACAGAUGUGUCUUGGAAGAGUCGGAGAGU			ND	NA	NC	NC
MENC_100	B230339C10	GCAGUUCUGACAUUUUGGUGUGUUUCAUUGUUGGGCUAUGCACAGAAUGUUCCUACUGUCUU			ND	NA	NC	C
MENC_101	B230399F14	UUUGUUUUUCCUGCUUCCUUUUCCCCAUGGAGCGUGGGGAGCAGGCCUCAGAGCAAAGU			ND	NA	NC	C
MENC_102	B230206C19						NC	

[a] : Nmir_XXX indicates predicted miRNAs which have not yet been registered in miRBase 8.0

[b] : D: detected; ND: not detected; NA: not applicable

[c] : C: conserved between mouse and human; NC: not conserved between mouse and human;

Table S3: List of probes for microarray

PROBE_1	TTTTTTTTTTTTTTTTTACCCGACCAACAGCACAAACTACTAC
PROBE_2	TTTTTTTTTTTTTTTTTAGGCAGTAGCTTGCGCAGTTATCTCC
PROBE_3	TTTTTTTTTTTTTTTTTTTTTAAATCAAGGTCCGCTGTGAACA
PROBE_4	TTTTTTTTTTTTTTTTTTTTTGGCATTCACCGCGTGCCTTAAT
PROBE_5	TTTTTTTTTTTTTTTTTACAGTAGCACAATGTGAAAAGAGCTC
PROBE_6	TTTTTTTTTTTTTTTTTCGATGCCCTTTTAACATTGCACTGCT
PROBE_7	TTTTTTTTTTTTTTTTTACTTACAGTATATGATGATATCCCAG
PROBE_8	TTTTTTTTTTTTTTTTTGACTAGTACATCATCTATACTGTAGT
PROBE_9	TTTTTTTTTTTTTTTTTGTAATGGTAACGGTTTCCTCGCCATT
PROBE_10	TTTTTTTTTTTTTTTTTGGAGCAGCAAGAGAACCGTTACCATT
PROBE_11	TTTTTTTTTTTTTTTTTAACAGCTGCTTTTGGGATTCCGTTGC
PROBE_12	TTTTTTTTTTTTTTTTTAGGGAACGAAATCCAAGTGCAGCTGG
PROBE_13	TTTTTTTTTTTTTTTTTTTCCACAAAGAAGTATATGCATAGGA
PROBE_14	TTTTTTTTTTTTTTTTTCATCTTCCCATGCGCTATACCTCTTT
PROBE_15	TTTTTTTTTTTTTTTTTGACAGACTCCGGTGGAATGAAGGACA
PROBE_16	TTTTTTTTTTTTTTTTTTTCTGAGCTTCACTCCACTGAAATCT
PROBE_17	TTTTTTTTTTTTTTTTTTTCGGACTTGGTTCCGTTTGACCAGC
PROBE_18	TTTTTTTTTTTTTTTTTTTGTAGCTGGTTGAAGGGGACCAAAC
PROBE_19	TTTTTTTTTTTTTTTTTTTCTATGAGGATATAAAGAAGCATGT
PROBE_20	TTTTTTTTTTTTTTTTTTTCCACACACTTCCTTACATTCCATA

PROBE_21	TTTTTTTTTTTTTTTTTTAAAACTATGCAACCTACTACCTCT
PROBE_22	TTTTTTTTTTTTTTTTAGGCAGCAGGTCGTATAGTTAACTCC
PROBE_23	TTTTTTTTTTTTTTTTTTCATAAAGCTTGCCACTGAAGAACT
PROBE_24	TTTTTTTTTTTTTTTTCAACAGTTCTTCAACTGGCAGCTTTA
PROBE_25	TTTTTTTTTTTTTTTTTTGGATCCCTGGGACCATACCTAGAA
PROBE_26	TTTTTTTTTTTTTTTTTGGTTCTAGGATAGGCCCAGGGGCCT
PROBE_27	TTTTTTTTTTTTTTTTCTGCACAGCAAGTGTAGACAGGCAGA
PROBE_28	TTTTTTTTTTTTTTTTTGACTGCCTGTCTGTGCCTGCTGTAC
PROBE_29	TTTTTTTTTTTTTTTTTGTCCAACTCAGCTTGTCAAATACAC
PROBE_30	TTTTTTTTTTTTTTTTTGGGGTATTTGACAAACTGACACTCT
PROBE_31	TTTTTTTTTTTTTTTTTTGCGAATTCACCAAGGGCAACCTCT
PROBE_32	TTTTTTTTTTTTTTTTAAACAAAAGTTGCCTTTGTGTGATTC
PROBE_33	TTTTTTTTTTTTTTTTACACACAGGACCTGGAGTCAGGAGCC
PROBE_34	TTTTTTTTTTTTTTTTAGGCCTTCTGACTCCAAGTCCAGTGC
PROBE_35	TTTTTTTTTTTTTTTTAAAGTCTCGCTCTCTGCCCCTCAGCC
PROBE_36	TTTTTTTTTTTTTTTTAAGACTGAGGGGCCTCAGACCGAGCT
PROBE_37	TTTTTTTTTTTTTTTTTACAAAACTTTGGTCAAGAGTAGAAA
PROBE_38	TTTTTTTTTTTTTTTTTTATCCTCCCTCCCGTCCCCAAATTC
PROBE_39	TTTTTTTTTTTTTTTTCCTCCAGCCCCTCCAGGGCTTCCTCC
PROBE_40	TTTTTTTTTTTTTTTTGAGGTGGAGCCCTGAGAACCGGAGGA
PROBE_41	TTTTTTTTTTTTTTTTTCATCTCAGGCGCAGCAGATACCTCT

PROBE_42	TTTTTTTTTTTTTTTTTTCGAGGCGTCCAGCGCCCGCGCCCG
PROBE_43	TTTTTTTTTTTTTTTTGAAGAGGCAGGTGGTAGGGGAGAGAG
PROBE_44	TTTTTTTTTTTTTTTTCCCTCCCTTCACCCTCTCCCTTGCCT
PROBE_45	TTTTTTTTTTTTTTTTGCAACGGCCCCGCGCTGCCGGCGCTG
PROBE_46	TTTTTTTTTTTTTTTTACCAACGCCAGAGAGATGGGGCTCTA
PROBE_47	TTTTTTTTTTTTTTTTAAAGCTACAAGTACGACAAGGCGATC
PROBE_48	TTTTTTTTTTTTTTTTCGCATCGCCCCGGGAGGCTCCCCGCA
PROBE_49	TTTTTTTTTTTTTTTTCAACCCGGCCAAGAAGAAGAGGAAAA
PROBE_50	TTTTTTTTTTTTTTTTCCTCCTCCTCGTCCTCCTCGGGGGTA
PROBE_51	TTTTTTTTTTTTTTTTAAAGACATCACTGTAATGAGTGTGGG
PROBE_52	TTTTTTTTTTTTTTTTTCCTCCTCACCCACCATCAGAGAGTC
PROBE_53	TTTTTTTTTTTTTTTTTCCCCCCTCTTTCTCCATCCAAAGCA
PROBE_54	TTTTTTTTTTTTTTTTTCCTCACCGGACAGAACGAGTTGAAG
PROBE_55	TTTTTTTTTTTTTTTTCGGGAAATAAAGCCGGCCCCCGCGGA
PROBE_56	TTTTTTTTTTTTTTTTATTCCCGGGGGTGACTCCTTTCTAGT
PROBE_57	TTTTTTTTTTTTTTTTTTGATCCCCTGTGAACCTTGACCTAT
PROBE_58	TTTTTTTTTTTTTTTTTTTTAGGTCAAAGTAAACAGATTATA
PROBE_59	TTTTTTTTTTTTTTTTGAGGAAGCAGATTCGGCCATCGCTGA
PROBE_60	TTTTTTTTTTTTTTTTAATCAACGGAATGAGCAGAATCTGAA
PROBE_61	TTTTTTTTTTTTTTTTCCAGGCTCCGCCCTCTTCTAGCTTCC
PROBE_62	TTTTTTTTTTTTTTTTATTGAAGGACCAGAGAGGGCGGCCCG

PROBE_63	TTTTTTTTTTTTTTTTCCTCAACTCGTTCACTCAGGGAATCC
PROBE_64	TTTTTTTTTTTTTTTTCGGGACCCCCGGGAGTACGAGCGGCA
PROBE_65	TTTTTTTTTTTTTTTTGTCCAATTATACAGCAAACACTCCCA
PROBE_66	TTTTTTTTTTTTTTTTTCAAGTCATGCCTACTGCACCTCTAA
PROBE_67	TTTTTTTTTTTTTTTTCATGTCTCAGGAAGAAGAAGAAGGAG
PROBE_68	TTTTTTTTTTTTTTTTCCCACACTTGTTCCTCTTCCAGGAGC
PROBE_69	TTTTTTTTTTTTTTTTCCTTCATATTGCAGGGCCCGGCGGCG
PROBE_70	TTTTTTTTTTTTTTTTCATGCTCGGATGCCCGGTGCCCGCAC
PROBE_71	TTTTTTTTTTTTTTTTAACCTCCCTCCAGAGGAGCAGAACAG
PROBE_72	TTTTTTTTTTTTTTTTAAATGTTTAATCTACCTGAAGAGAGG
PROBE_73	TTTTTTTTTTTTTTTTCGACCATCAAACGCATCCAGCAGCAG
PROBE_74	TTTTTTTTTTTTTTTTCCCTACCGCGTGGTGGGACTGGTGGT
PROBE_75	TTTTTTTTTTTTTTTTCCCTGCCGGCAGGTAGCAGAGAAAAG
PROBE_76	TTTTTTTTTTTTTTTTGCCCCTCCTCTTCAGCCCGCTGGCGG
PROBE_77	TTTTTTTTTTTTTTTTCTCACAACAACCCTAGGAGGTTGCTC
PROBE_78	TTTTTTTTTTTTTTTTTTCAGCCCTGCCTCAGGCTGCTGCAG
PROBE_79	TTTTTTTTTTTTTTTTCCAGCAAGAGACAGCGAGGGGGAGTG
PROBE_80	TTTTTTTTTTTTTTTTCCACCTCCAGATCCTCTGCTGCTCCC
PROBE_81	TTTTTTTTTTTTTTTTACTGTGGGCCCTTTCCGCACCAGTCC
PROBE_82	TTTTTTTTTTTTTTTTAGGGACTGAGCGGTTAGGGCATACAG
PROBE_83	TTTTTTTTTTTTTTTTATCCGGGCTCTGGTGGCCTGAGGGGG

PROBE_84	TTTTTTTTTTTTTTTTGCTCCCCACAGACCCAGAGCCGAATT
PROBE_85	TTTTTTTTTTTTTTTTTTTGTGTTCACGTGGCAACCGTACCT
PROBE_86	TTTTTTTTTTTTTTTTTTTGAGGACGGCGCTAACCACATGCT
PROBE_87	TTTTTTTTTTTTTTTTTTCCCCGGCTCTCAGGAAGTGGTGGC
PROBE_88	TTTTTTTTTTTTTTTTTTCCGCCGCCTCGACTCCTCCGTGAC
PROBE_89	TTTTTTTTTTTTTTTTGGCCTTCTCAACTCTCATGAAAAAGG
PROBE_90	TTTTTTTTTTTTTTTTTTACCTTCTCATGAAGAAACAAAAGG
PROBE_91	TTTTTTTTTTTTTTTTGTCATGCAAAGCAATCACACATTCAT
PROBE_92	TTTTTTTTTTTTTTTTAGGAGAACATTGATGGCTCTGTTGAC
PROBE_93	TTTTTTTTTTTTTTTTGCTCCAACTGGGGCAGATCGGCGACA
PROBE_94	TTTTTTTTTTTTTTTTCATGTCGTCGATCATCTCGCCCACCT
PROBE_95	TTTTTTTTTTTTTTTTCTTACCATCTTCAAAGGGTGTCCCTT
PROBE_96	TTTTTTTTTTTTTTTTCAAAGGGCACCCTTTTAATATGTAGA
PROBE_97	TTTTTTTTTTTTTTTTTCTTGGCAAAAGACAACACCTCTCCC
PROBE_98	TTTTTTTTTTTTTTTTTGGGGCAGCCCTCTGCCTTCTGCCAA
PROBE_99	TTTTTTTTTTTTTTTTACAGTTCCCAGAGGGGTACCGATCGT
PROBE_100	TTTTTTTTTTTTTTTTCATGAATCAAACCACCAGCCTGAGCT
PROBE_101	TTTTTTTTTTTTTTTTGTAGATCTCTTCACTGGCCAGAAACC
PROBE_102	TTTTTTTTTTTTTTTTCAGATGCCTGGCAGTAACAGAGCTTC
PROBE_103	TTTTTTTTTTTTTTTTTCTTGGCCGCCTCTGGTCCAAGACTG
PROBE_104	TTTTTTTTTTTTTTTTTTCTATCCGGCACTGGCGGCTCCAAG

PROBE_105	TTTTTTTTTTTTTTTTAGACAGTGTGCAAACCTGATCTCCAG
PROBE_106	TTTTTTTTTTTTTTTTGGCGAGAGATCAGGATTCCTCACTGG
PROBE_107	TTTTTTTTTTTTTTTTCGTGGTGGGGACACACACACCGCAAC
PROBE_108	TTTTTTTTTTTTTTTTGGGTTGGGTTGTAGTCCGACCACCAC
PROBE_109	TTTTTTTTTTTTTTTTATCTCATTTGTCTTCTCACACTGG
PROBE_110	TTTTTTTTTTTTTTTTATGCAGAGCGAGAAAACAATACAGTT
PROBE_111	TTTTTTTTTTTTTTTTCCACAGACCAATCAGATGCAGGCAAT
PROBE_112	TTTTTTTTTTTTTTTTCTCTCGCCTCCATCAATTAGCTATCA
PROBE_113	TTTTTTTTTTTTTTTTAGAGTTCAAGCATAGGAGCCTATGGA
PROBE_114	TTTTTTTTTTTTTTTTAACCCACAGACTCCCAAAACAGTGCC
PROBE_115	TTTTTTTTTTTTTTTTGCCACAAGTCTGCCGGACTTCCTCAC
PROBE_116	TTTTTTTTTTTTTTTTAACGAGAACTCACCCAAGTCAAACT
PROBE_117	TTTTTTTTTTTTTTTTGTAGGAGCCAGGCTTCAGCCAGAGGC
PROBE_118	TTTTTTTTTTTTTTTTACAGCTGCAGCTGAAATCACTGGCCA
PROBE_119	TTTTTTTTTTTTTTTTCAGTGACAGAGGTGGGCTCCTGGGT
PROBE_120	TTTTTTTTTTTTTTTTAAACCCAAAGCTTGCAAGTGACACCT
PROBE_121	TTTTTTTTTTTTTTTTGATACCAGAATAGCAACAGCCACTTC
PROBE_122	TTTTTTTTTTTTTTTTATGAAGGGCTGTTGCTTAGTTTCTAC
PROBE_123	TTTTTTTTTTTTTTTTCAGGCCACTGGGCTGCAGGAAATGGG
PROBE_124	TTTTTTTTTTTTTTTTAGCCCGTCCTACATAACCCTGTGTAC
PROBE_125	TTTTTTTTTTTTTTTTATTGATTAGCACCATAGCTGAACCAA

PROBE_126	TTTTTTTTTTTTTTTTTAATGGCATTCAGCTATGGAACCTGA
PROBE_127	TTTTTTTTTTTTTTTTAGGCATATCCAGCAGTTCCCCAAACA
PROBE_128	TTTTTTTTTTTTTTTTGTTCTTTGGGGACTACCCAGGCAAGC
PROBE_129	TTTTTTTTTTTTTTTTAACTCAGCAAAGTACAGACATGCAGG
PROBE_130	TTTTTTTTTTTTTTTTTTTCAGCATGTTCTGACCACTGCTGC
PROBE_131	TTTTTTTTTTTTTTTTATTCTTTTGAAAGAAAACTCCCCTCT
PROBE_132	TTTTTTTTTTTTTTTTATGTGGGGGAGGGGTTTCCTTCAAAT
PROBE_133	TTTTTTTTTTTTTTTTCAAACAGCAGTGGGGTCAGGGGCAGG
PROBE_134	TTTTTTTTTTTTTTTTCACCTGCCCACTTCACCCCACTTAGC
PROBE_135	TTTTTTTTTTTTTTTTTAAAGGATTAAGGGAACCGGATAAAC
PROBE_136	TTTTTTTTTTTTTTTTTGATCTTCCAGCTCCCCTCACCCAAC
PROBE_137	TTTTTTTTTTTTTTTTCTTTAAAATGGGCCAAGTGGCTCAGA
PROBE_138	TTTTTTTTTTTTTTTTTGCATGATGAACTTGGTCCCAATTTT
PROBE_139	TTTTTTTTTTTTTTTTGGCTGTAGGCGGAGCAGCTCATGGGC
PROBE_140	TTTTTTTTTTTTTTTTGGCACCAGGAGCTGCTCACCGCGCAG
PROBE_141	TTTTTTTTTTTTTTTTTCCAGGATTTTCCAGCCCAGACCCTA
PROBE_142	TTTTTTTTTTTTTTTTATCAAGAGTCTGAACTGGACCTGAAC
PROBE_143	TTTTTTTTTTTTTTTTGATCATTTCCATGAGGCCCAGGCGTA
PROBE_144	TTTTTTTTTTTTTTTTTGGACACCTGGCCCTTACAGAAGATC
PROBE_145	TTTTTTTTTTTTTTTTACAGAGGCCAGGCAGACCAAGAAACT
PROBE_146	TTTTTTTTTTTTTTTTTTTGTTGCCTTAGCATGCCTGATAGC

PROBE_147	TTTTTTTTTTTTTTTTCAACCATTATTGGCATCAAACACAGT
PROBE_148	TTTTTTTTTTTTTTTTACAAAGAGGGTCTGATGCCAACCTCG
PROBE_149	TTTTTTTTTTTTTTTTTAGATCCACCATCTTTGTATGTGAGC
PROBE_150	TTTTTTTTTTTTTTTTTGTCTCACACACAAAGAAGTGGGACT
PROBE_151	TTTTTTTTTTTTTTTTTTTGCCTACATACACATCACACACAC
PROBE_152	TTTTTTTTTTTTTTTTATGTGTATGCACCTGTGTATGTATAA
PROBE_153	TTTTTTTTTTTTTTTTAGCAGGTGGCTCCTTCCCAAGCATAT
PROBE_154	TTTTTTTTTTTTTTTTTTCTATGCTGGGAACAGGAAGTCCAC
PROBE_155	TTTTTTTTTTTTTTTTAGTCAGACTGCAGAGGAGACAAAAGA
PROBE_156	TTTTTTTTTTTTTTTTCTTACTCTCTCTCCTTTGTCCTCATC
PROBE_157	TTTTTTTTTTTTTTTTGGTAGTGATGGAGGTGAGCCTTTTTC
PROBE_158	TTTTTTTTTTTTTTTTCCGTAAAAGTGCTTTCCTCCATCCCA
PROBE_159	TTTTTTTTTTTTTTTTTAAATCCGAAGGGAAATGAGCAAACC
PROBE_160	TTTTTTTTTTTTTTTTTTCGCTCGCCCACCTCTTCCAAATTT
PROBE_161	TTTTTTTTTTTTTTTTAGATGAGCCAATTCAATCCTCGCACC
PROBE_162	TTTTTTTTTTTTTTTTCATCTGCAAGGATCCCATTGATGCCA
PROBE_163	TTTTTTTTTTTTTTTTCTCTGTATCTTGGAAGTTTGCCGAGT
PROBE_164	TTTTTTTTTTTTTTTTGCACTCTCCGACTCTTCCAAGACACA
PROBE_165	TTTTTTTTTTTTTTTTAACACACCAAAATGTCAGAACTGCTT
PROBE_166	TTTTTTTTTTTTTTTTCAAAGACAGTAGGAACATTCTGTGCA
PROBE_167	TTTTTTTTTTTTTTTTGGAAAAGGAAGCAGGAAAAACAAAAC

PROBE_168	TTTTTTTTTTTTTTTTAGACTTTGCTCTGAGGCCCTGCTCCC

Table S4: List of Stem-loop RT-PCR primers

all miRNAs	Reverse primer	GTGCAGGGTCCGAGGT
mmu-let-7a	RT primer	GTCGTATCCAGTGCAGGGTCCGAGGTATTCGCACTGGATACGACACTATA
mmu-let-7a	Forward primer	GCCCTGAGGTAGTAGGTTG
Nmir_001	RT primer	GTCGTATCCAGTGCAGGGTCCGAGGTATTCGCACTGGATACGACTTACAG
Nmir_001	Forward primer	GCCCGGGATATCATCATATA
Nmir_002	RT primer	GTCGTATCCAGTGCAGGGTCCGAGGTATTCGCACTGGATACGACGCACAG
Nmir_002	Forward primer	GCCCTGCCTGTCTACACTTG
Nmir_004	RT primer	GTCGTATCCAGTGCAGGGTCCGAGGTATTCGCACTGGATACGACAGTCTC
Nmir_004	Forward primer	GCCCCTGAGGGGCAGAGAGC
Nmir_006	RT primer	GTCGTATCCAGTGCAGGGTCCGAGGTATTCGCACTGGATACGACATCCTC
Nmir_006	Forward primer	GCCCATTTGGGGACGGGAGG
Nmir_008	RT primer	GTCGTATCCAGTGCAGGGTCCGAGGTATTCGCACTGGATACGACCGAGGC
Nmir_008	Forward primer	GCCCGGCGCGGGCGCTGGAC
Nmir_009	RT primer	GTCGTATCCAGTGCAGGGTCCGAGGTATTCGCACTGGATACGACAGAGGC
Nmir_009	Forward primer	GCCCCTCTCCCCTACCACCT
Nmir_010	RT primer	GTCGTATCCAGTGCAGGGTCCGAGGTATTCGCACTGGATACGACCTCCCT
Nmir_010	Forward primer	GCCCGCAAGGGAGAGGGTGA
Nmir_011	RT primer	GTCGTATCCAGTGCAGGGTCCGAGGTATTCGCACTGGATACGACAACGGC
Nmir_011	Forward primer	GCCCGCGCCGGCAGCGCGGG
Nmir_012	RT primer	GTCGTATCCAGTGCAGGGTCCGAGGTATTCGCACTGGATACGACCATCGC

Nmir_012	Forward primer	GCCCCGGGGAGCCTCCCGGG
Nmir_013	RT primer	GTCGTATCCAGTGCAGGGTCCGAGGTATTCGCACTGGATACGACTCCTCC
Nmir_013	Forward primer	GCCCCCCCCGAGGAGGACGA
Nmir_014	RT primer	GTCGTATCCAGTGCAGGGTCCGAGGTATTCGCACTGGATACGACCTCCTC
Nmir_014	Forward primer	GCCCCTCTCTGATGGTGGGT
Nmir_015	RT primer	GTCGTATCCAGTGCAGGGTCCGAGGTATTCGCACTGGATACGACCCCCCT
Nmir_015	Forward primer	GCCCCTTTGGATGGAGAAAG
Nmir_016	RT primer	GTCGTATCCAGTGCAGGGTCCGAGGTATTCGCACTGGATACGACCTCACC
Nmir_016	Forward primer	GCCCTCAACTCGTTCTGTCC
Nmir_017	RT primer	GTCGTATCCAGTGCAGGGTCCGAGGTATTCGCACTGGATACGACGGAAAT
Nmir_017	Forward primer	GCCCCGCGGGGGCCGGCTTT
Nmir_018	RT primer	GTCGTATCCAGTGCAGGGTCCGAGGTATTCGCACTGGATACGACGATCCC
Nmir_018	Forward primer	GCCCAGGTCAAGGTTCACAG
Nmir_019	RT primer	GTCGTATCCAGTGCAGGGTCCGAGGTATTCGCACTGGATACGACGGAAGC
Nmir_019	Forward primer	GCCCAGCGATGGCCGAATCT
Nmir_020	RT primer	GTCGTATCCAGTGCAGGGTCCGAGGTATTCGCACTGGATACGACTGAAGG
Nmir_020	Forward primer	GCCCGGCCGCCCTCTCTGGT
Nmir_021	RT primer	GTCGTATCCAGTGCAGGGTCCGAGGTATTCGCACTGGATACGACGGACCC
Nmir_021	Forward primer	GCCCCCGCTCGTACTCCCGG
Nmir_022	RT primer	GTCGTATCCAGTGCAGGGTCCGAGGTATTCGCACTGGATACGACAAGTCA
Nmir_022	Forward primer	GCCCAGAGGTGCAGTAGGCA

Nmir_023	RT primer	GTCGTATCCAGTGCAGGGTCCGAGGTATTCGCACTGGATACGACTGTCTC
Nmir_023	Forward primer	GCCCCCTTCTTCTTCTTCCT
Nmir_024	RT primer	GTCGTATCCAGTGCAGGGTCCGAGGTATTCGCACTGGATACGACTTCATA
Nmir_024	Forward primer	GCCCCCGCCGGGCCCTGCAA
Nmir_025	RT primer	GTCGTATCCAGTGCAGGGTCCGAGGTATTCGCACTGGATACGACTGCTCG
Nmir_025	Forward primer	GCCCGCGGGCACCGGGCATC
Nmir_026	RT primer	GTCGTATCCAGTGCAGGGTCCGAGGTATTCGCACTGGATACGACCCTCCC
Nmir_026	Forward primer	GCCCGTTCTGCTCCTCTGGA
Nmir_027	RT primer	GTCGTATCCAGTGCAGGGTCCGAGGTATTCGCACTGGATACGACCTACCG
Nmir_027	Forward primer	GCCCCACCAGTCCCACCACG
Nmir_028	RT primer	GTCGTATCCAGTGCAGGGTCCGAGGTATTCGCACTGGATACGACCCCTCC
Nmir_028	Forward primer	GCCCGCCAGCGGGCTGAAGA
Nmir_029	RT primer	GTCGTATCCAGTGCAGGGTCCGAGGTATTCGCACTGGATACGACAGCCCT
Nmir_029	Forward primer	GCCCTGCAGCAGCCTGAGGC
Nmir_030	RT primer	GTCGTATCCAGTGCAGGGTCCGAGGTATTCGCACTGGATACGACACCTCC
Nmir_030	Forward primer	GCCCGAGCAGCAGAGGATCT
Nmir_003: mmu-miR-422b	RT primer	GTCGTATCCAGTGCAGGGTCCGAGGTATTCGCACTGGATACGACGGCCTT
Nmir_003: mmu-miR-422b	Forward primer	GCCCCTGGACTTGGAGTCAG
Nmir_005: mmu-mir-423	RT primer	GTCGTATCCAGTGCAGGGTCCGAGGTATTCGCACTGGATACGACCTGAGG
Nmir_005: mmu-mir-423	Forward primer	GCCCAGCTCGGTCTGAGGCC
Nmir_007: mmu-miR-671	RT primer	GTCGTATCCAGTGCAGGGTCCGAGGTATTCGCACTGGATACGACCCTCCA

Nmir_007: mmu-miR-671	Forward primer	GCCCAGGAAGCCCTGGAGGGGC
Nmir_031: mmu-miR-675-3p	RT primer	GTCGTATCCAGTGCAGGGTCCGAGGTATTCGCACTGGATACGACACTGAG
Nmir_031: mmu-miR-675-3p	Forward primer	GCCCCTGTATGCCCTAACCG
Nmir_032: mmu-miR-760	RT primer	GTCGTATCCAGTGCAGGGTCCGAGGTATTCGCACTGGATACGACCTCCCC
Nmir_032: mmu-miR-760	Forward primer	GCCCGAAATTCGGCTCTGGGTCTGT
